# Modulating cell stiffness for improved vascularization: leveraging the MIL-53(fe) for improved interaction of titanium implant and endothelial cell

**DOI:** 10.1186/s12951-024-02714-y

**Published:** 2024-07-17

**Authors:** Jie Wu, Leyi Liu, Weidong Du, Yunyang Lu, Runze Li, Chao Wang, Duoling Xu, Weili Ku, Shujun Li, Wentao Hou, Dongsheng Yu, Wei Zhao

**Affiliations:** 1 Hospital of Stomatology, Guanghua School of Stomatology, Sun Yat-sen University, Guangzhou, 510055 China; 2 Guangdong Provincial Key Laboratory of Stomatology, Sun Yat-sen University, Guangzhou, 510050 China; 3 Institute of Metal Research, Chinese Academy of Sciences, Shenyang, 110016 China

**Keywords:** Vascularization, MIL-53(Fe), Endothelial tip cell, Mechanotransduction, Cell stiffness

## Abstract

Vascularization plays a significant role in promoting the expedited process of bone regeneration while also enhancing the stability and viability of artificial bone implants. Although titanium alloy scaffolds were designed to mimic the porous structure of human bone tissues to facilitate vascularization in bone repair, their biological inertness restricted their broader utilization. The unique attribute of Metal-organic framework (MOF) MIL-53(Fe), known as “breathing”, can facilitate the efficient adsorption of extracellular matrix proteins and thus provide the possibility for efficient interaction between scaffolds and cell adhesion molecules, which helps improve the bioactivity of the titanium alloy scaffolds. In this study, MIL-53(Fe) was synthesized in situ on the scaffold after hydrothermal treatment. The MIL-53(Fe) endowed the scaffold with superior protein absorption ability and preferable biocompatibility. The scaffolds have been shown to possess favorable osteogenesis and angiogenesis inducibility. It was indicated that MIL-53(Fe) modulated the mechanotransduction process of endothelial cells and induced increased cell stiffness by promoting the adsorption of adhesion-mediating extracellular matrix proteins to the scaffold, such as laminin, fibronectin, and perlecan et al., which contributed to the activation of the endothelial tip cell phenotype at sprouting angiogenesis. Therefore, this study effectively leveraged the intrinsic “breathing” properties of MIL-53 (Fe) to enhance the interaction between titanium alloy scaffolds and vascular endothelial cells, thereby facilitating the vascularization inducibility of the scaffold, particularly during the sprouting angiogenesis phase. This study indicates that MIL-53(Fe) coating represents a promising strategy to facilitate accelerated and sufficient vascularization and uncovers the scaffold-vessel interaction from a biomechanical perspective.

**Graphical Abstract:**

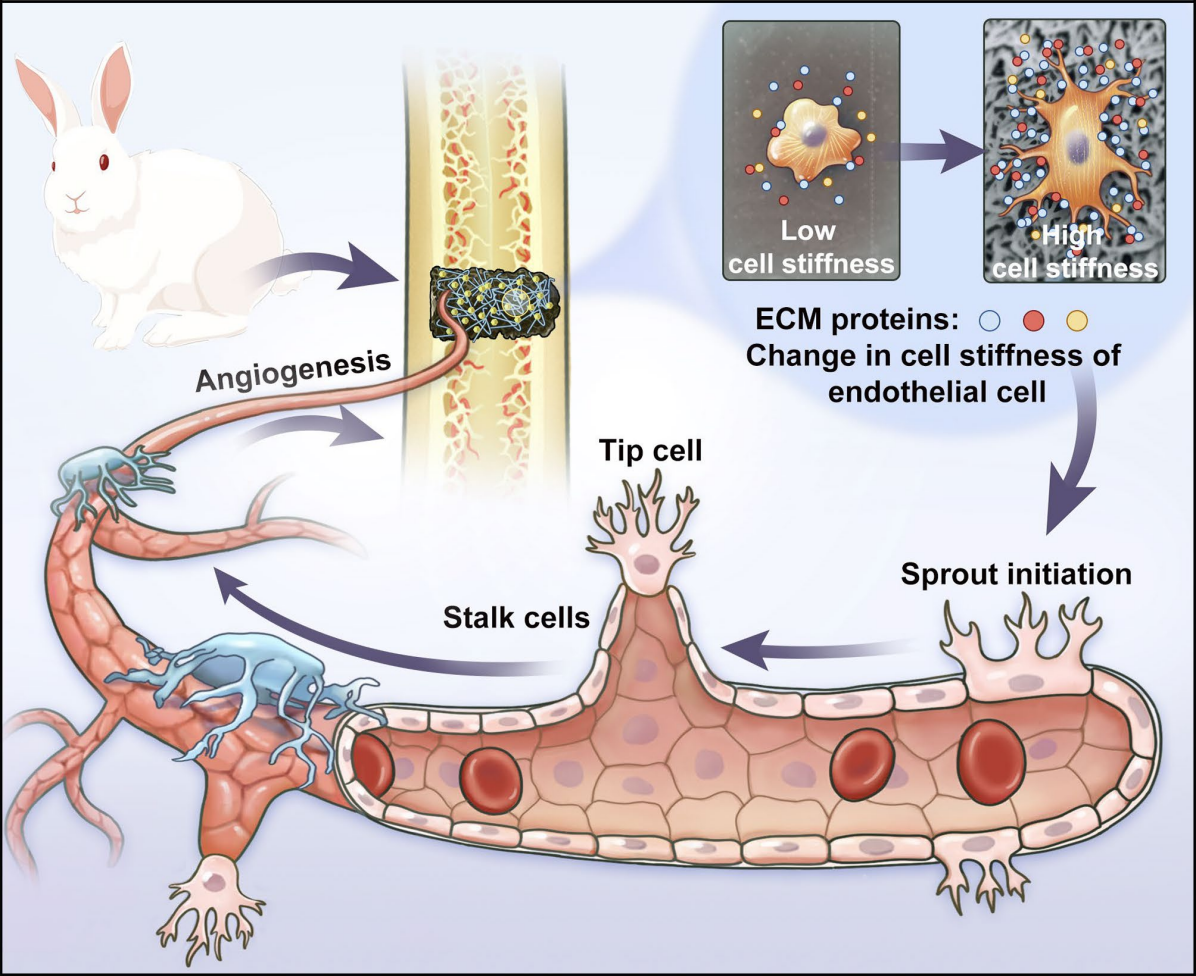

**Supplementary Information:**

The online version contains supplementary material available at 10.1186/s12951-024-02714-y.

## Introduction

Angiogenesis, the growth of new vessels from the pre-existing blood vasculature, is essential to bone development and remodeling [1]. On the one hand, it is beneficial to create an optimal microenvironment for accelerated bone defect reconstruction by facilitating rapid blood vessel growth [2]; on the other hand, sufficient vascularization favors sustained osteogenic activity of osteoblasts, thereby promoting survival of the scaffold [3]. However, insufficient vascularization of artificial bone repair material remains a challenging problem [4, 5]. The porous titanium scaffold, manufactured to mimic natural bone, was characterized by stimulating osteogenesis and vascularization [6]. Nevertheless, the bioinert surface of titanium alloy limits its angiogenic and osteogenic induction [7, 8].

The growth of the vascular system undergoes activation of endothelial cells (ECs) differentiation to tip cells, sprout formation by proliferating stalk cells, and ultimate stabilization of vascularization [9]. Tip cell, a specialized EC at the distal end of each sprout, plays a significant part in spearheading new sprouts and navigating the extension of new blood vessels. After activation of the tip cell, the adjacent stalk cells will be facilitated to proliferate and elongate [10]. Moreover, endothelial tip cells can also join with the ones of the adjacent sprouting blood vessels to form tube lumens [11]. However, few researches focus on the effect of artificial bone grafts on specific stages of angiogenesis, in particular, the stages of sprouting angiogenesis and activation of endothelial tip cells.

In addition to being manipulated by biochemical molecules like vascular endothelial growth factor (*VEGF*) [12], vascular endothelial cells are also affected by biomechanical signals during the angiogenesis process [13]. Integrins are the critical cellular structures responsible for mechanosensing and enabling the attachment of cells to the extracellular matrix (ECM). As the forces associated with sensing ECM viscoelasticity at cell-ECM contacts propagate to the actin cytoskeleton, they are finally propagated to chromatin via the linker of nucleoskeleton and cytoskeleton (LINC) that bridge cytoplasmic actin with nuclear lamins, which will influence the gene expressions and cell behaviors [14]. The process by which cells convert mechanical signals to biochemical signals is called mechanotransduction. A prominent biomechanical manifestation of mechanotransduction is the alteration of cell stiffness [15]. Cell stiffness is mainly determined by the cholesterol content of the plasma membrane [16] and the underlying actin cortex of the cortical structures [17]. This study aimed to investigate the mechanisms by which artificial bone repair scaffolds promoted vascularization from the perspective of cellular stiffness regulation.

Metal-organic Framework (MOF), constructed from metal ion/cluster nodes and functional organic ligands through coordination bonds, have the advantages of facile synthesis, large surface areas, adjustable porosity, and improved biosafety, which have been widely used in biomedical fields [18]. Nanoscale MOF has also been applied in artificial bone repair scaffolds for their controllable release of bioactive ions, drugs or exosomes [19–22]. MIL-53(Fe), a subclass of Fe-MOF consisting of iron (Fe) ions and terephthalic acid, possesses chemical stability, low toxicity, and peroxidase-like catalytic activity [23]. It has been reported that MIL-53(Fe) shows good stability and almost no iron ions leaching from it in the aqueous solution [24, 25]. Additionally, a unique characteristic called “breathing” given its flexible framework, allows it to adapt its porosity and optimize guest cargo-matrix interactions thus maximizing combination interactions and minimizing steric hindrance [26, 27]. This feature may enable MIL-53(Fe) to be a promising candidate for improving the bioactivity of titanium scaffold, which will play an important role in adsorbing various ECM proteins, resulting in facilitating the interactions of cells and scaffolds.

**Fig. 1:**
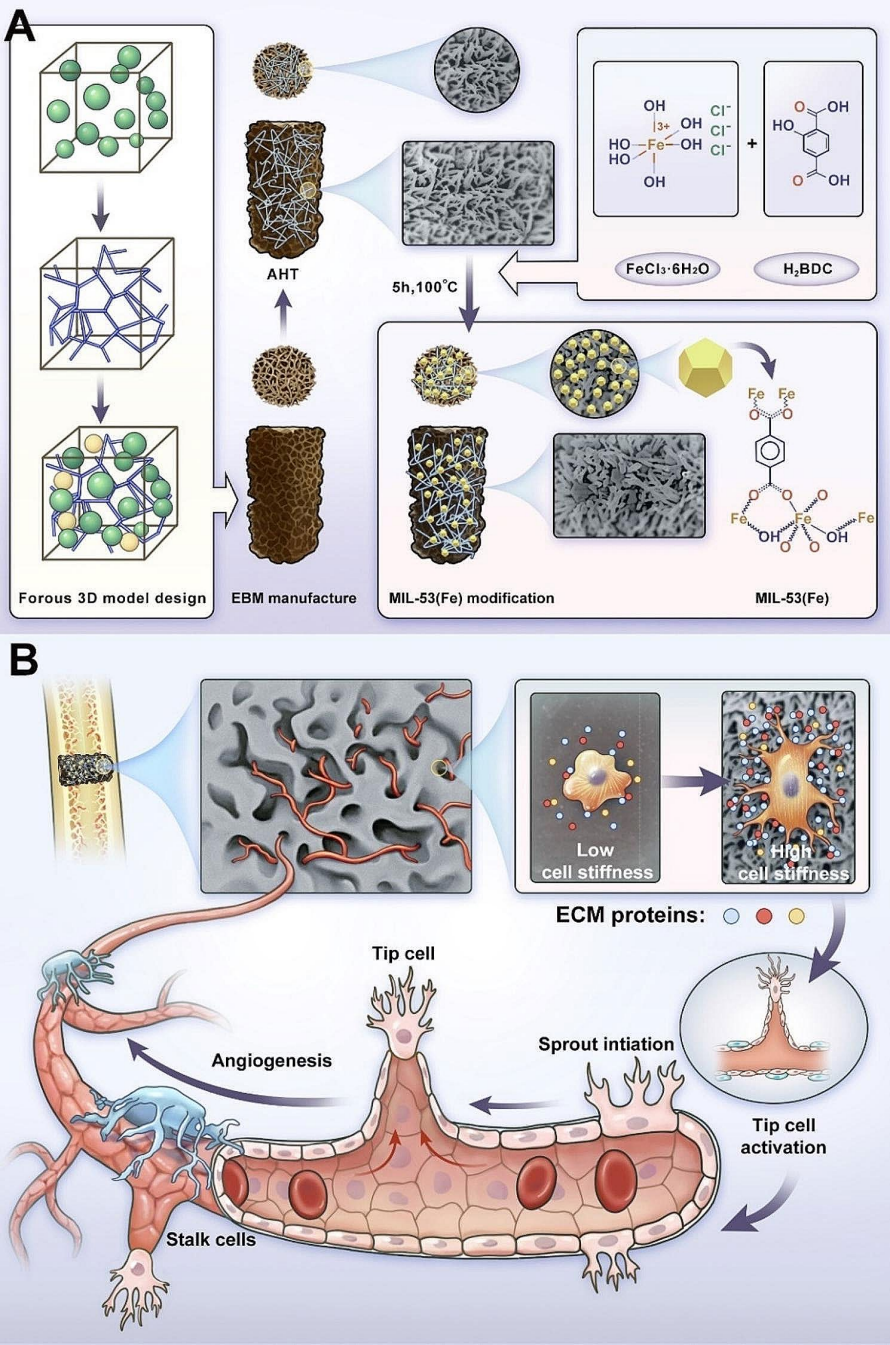
Schematic illustration of this study. (**A**) MIL-53(Fe) coating was constructed on the AHT surface of a 3D-printed bionic porous Ti-6Al-4V scaffold for enhanced vascularization in bone repair. (**B**) The MIL-53(Fe) modified scaffold was capable of promoting the adsorption of ECM proteins, which induce increased cell stiffness of vascular endothelial cells, thereby facilitating activation of endothelial tip cells in spouting angiogenesis and boosting sufficient vascularization in bone regeneration

Herein, a nanoscale structure on the 3D-printed porous Ti-6Al-4 V scaffold was fabricated by in situ crystal growth of MIL-53(Fe) applied on the prepared alkali and heat titanium (AHT) coating. The MIL-53(Fe) modified Ti-6Al-4 V scaffolds presented satisfying biocompatibility and favorable osteogenic and angiogenic capability. The MIL-53(Fe)-coated implants could promote the activation of vascular endothelial tip cells in the spouting angiogenesis. Furthermore, it was uncovered that the “breathing” property of MIL-53(Fe) was conductive to adsorbing ECM proteins, such as laminin, fibronectin, and perlecan, which promoted the interaction between ECs and scaffolds. The intensive interaction facilitated the mechanotransduction process and enhanced the cell stiffness of ECs, which was beneficial to the activation of tip cells and accelerated angiogenesis. This study helps to provide a basis for the application of MIL-53(Fe) to promote accelerated and sufficient vascularization of titanium scaffold and reveal the effects of its biological interactions on angiogenesis from the biomechanical perspective (Fig. 1).

## Materials and methods

### The fabrication of porous 3D-printed Ti-6Al-4 V scaffolds

**Fig. 2:**
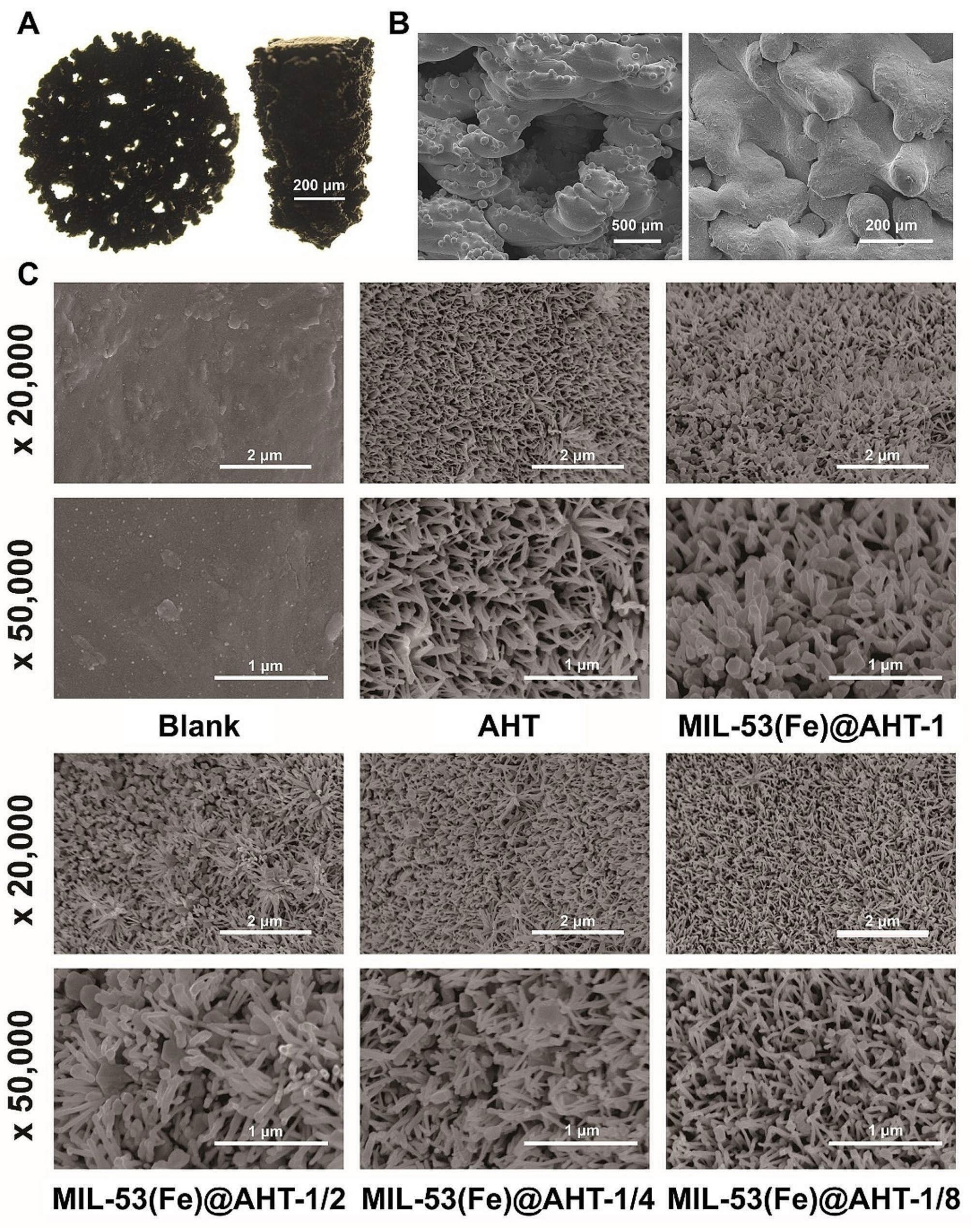
Morphological observation of the scaffolds. (**A**) The optical images of the 3D-printed Ti-6Al-4 V scaffolds for in vitro (Left) and in vivo (Right) experiments. (**B**) The SEM images of the scaffolds at the microscale. (**C**) The nano-topographic images of the scaffolds

The bionic porous Ti-6Al-4 V scaffolds were fabricated by an electron beam melting (EBM) system (ARCAM A1, Sweden), as mentioned previously [6]. The scaffolds with a diameter of 10 mm and thickness of 2 mm were used for in vitro studies, and the scaffolds for in vivo studies were 8 mm in height and 5 mm in diameter (Fig. 2A).

### Preparation of MIL-53(fe)@AHT structure

AHT was constructed as mentioned previously [28]. MIL-53(Fe) was fabricated using a solvothermal method, as reported in the literature [29]. Different densities were achieved by adjusting the synthesis solution concentrations. More specifically, the mixture of 1.35 g Ferric chloride hexahydrate (FeCl_3_·6H_2_O, Alfa, USA) and 0.83 g terephthalic acid (H_2_BDC, Alfa, USA) and 25 mL N, N dimethyl-formamide (DMF, Alfa, USA) were stirred for 2 h at 300 rpm. The mother solutions obtained were diluted to 1/2, 1/4, and 1/8 concentrations. The scaffolds of the AHT group, dipped horizontally into a Teflon autoclave and immersed in the solution, were heated for 5 h at 100 °C. The prepared scaffolds were termed as MIL-53(Fe)@AHT-1, MIL-53(Fe)@AHT-1/2, MIL-53(Fe)@AHT-1/4, MIL-53(Fe)@AHT-1/8, “1”, “1/2”, “1/4” and “1/8” indicating the concentration of the synthesis solutions. The precipitates, obtained by centrifuging the mother solution at 5000 rpm, were prepared for the following experiments.

### Scaffold characterization

The gross morphology of the scaffolds was observed by stereomicroscope (Leica MZ10 F, Germany). The surface topography was visualized by scanning electron microscope (SEM) at 20 kV (Nexsa, Thermo Fisher Scientific, USA). Surface roughness average (Ra) at micro and nano scale was evaluated by a confocal laser scanning microscopy (CLSM) (LSM700, Zeiss, Germany) and atomic force microscopy (AFM) (Dimension Fastscan Bio, Bruker, Germany) respectively. The wettability of the scaffolds was determined by contact angles of dropping distilled water using contact angle goniometer (OCAH200, Dataphyscics, Germany). XRD data of the precipitates were obtained using an X-ray powder diffraction (XRD) diffractometer (XRD, Supernova, Japan). The Fourier transform infrared (FTIR) data of the precipitates were obtained by an FTIR spectrometer (Nicolet NXR 9650, Thermo-Fisher, USA) ranging from 500 to 4000 cm^− 1^ with a potassium bromide disk.

### Protein absorption experiment

The Bovine serum albumin (BSA) (GIBCO, USA), and Fluorescein isothiocyanate (FITC)-conjugated Fetal bovine serum (FBS) (GIBCO, USA), laminin (Sigma-Aldrich, USA), fibronectin (MCE, USA) and perlecan (Sigma-Aldrich, USA) were used as the targeted proteins. The samples were incubated with 1 mL protein solution. In order to measure the absorption of BSA, the scaffolds were eluted with a 2% sodium dodecyl sulfonate solution to collect the absorbed BSA for protein quantitative analysis (BCA Protein Assay Kit, Pierce, USA). With CLSM (LSM 980, Carl Zeiss, Germany), samples incubated with FITC-FBS, FITC-laminin, FITC-fibronectin or FITC- perlecan were visualized.

### Cell culture

The human bone marrow stromal cells (hBMSCs) were obtained from Procell Life Science & Technology Co. (Wuhan, China) and the human umbilical vein endothelial cells (HUVECs) were purchased from ScienCell™ Research Laboratories (USA). Both kinds of cells were cultured in α-MEM (Gibco, USA) containing 10% (v/v) FBS (Gibco, USA) and 1% (v/v) penicillin/streptomycin (Gibco, USA). After reaching 80%, the cells were subjected to digestion by 0.25% trypsin-EDTA solution (Gibco, USA). For the subsequent experiments, hBMSCs from passages 2 to 3 and HUVECs from passages 2 to 5 were used.

Evaluation of cell viability, proliferation, attachment, spreading and morphology.

The hBMSCs (2 × 10^4^) and HUVECs (2 × 10^4^) were seeded on the substrate in a 24-well plate. As for the Cell Counting Kit-8 (CCK-8) assay, after incubation for 1, 3, 5 and 7 days, the cells were incubated with the medium containing 10% v/v CCK-8 solution (Dojindo Laboratories, Kumamoto, Japan) for 1 h in the dark at 37 ℃. The cell proliferation was examined by measuring the absorbance at 450 nm using a spectrophotometer (Bio-Tek, UK). In terms of live/dead staining assay, after 3 days of cell culture, calcein AM and ethidium homodimer-1 in the live/dead assay (Invitrogen, USA) were added in each well and incubated for 30 min at 37 ℃, which were evaluated by a CLSM (LSM780, Zeiss, Germany).

After culturing for 24 h, the cells (2 × 10^4^) on the scaffolds were incubated with the primary antibodies anti- integrin β1 (Itg β1) (1:200, Santa Cruz, USA) at 4 ℃ overnight, incubated with IgG-Cy3.5 secondary antibody (1:200, EMAR, China) at room temperature (RT) for 1 h, stained by the FITC-phalloidin (Solarbio, China) and DAPI (Solarbio, China) in sequence. The images of the scaffolds were recorded by CLSM (LSM 980, Carl Zeiss, Germany).

After incubation for 3 days, the cells (2 × 10^4^) on the substrates were fixed with 2.5% glutaraldehyde at 4°C for 24 h. A graded concentration of ethanol series (30, 50, 75, 90, 95, and 100% 10 min at each gradient) was used for cell dehydration. The morphology of the cells was observed by SEM (Nexsa, Thermo Fisher Scientific, USA).

### Preparation of sample conditioned medium

To prepare the sample conditioned medium, hBMSCs or HUVECs seeded on the scaffolds were incubated with the corresponding complete culture medium for 72 h. Filtered with a 0.22 μm filter (Millipore, USA), the sample conditioned medium was stored at 4 °C for subsequent experiments.

### Osteogenesis evaluation of hBMSCs

After cultivation with osteogenic medium for 7 days, the Alkaline phosphatase (ALP) staining of hBMSCs from different scaffolds was evaluated by the BCIP/NBT ALP color development kit (Beyotime, China). The ALP activity measurement kit (Jiancheng Nanjing, China) was used to analyze the ALP activity of hBMSCs, which was examined by measuring the absorbance at 450 nm.

After osteogenic induction for 21 days, the ECM mineralization of hBMSCs from different scaffolds was identified by Alizarin Red S (ARS) staining. The calcium deposition dissolved by 10% cetylpyridinium chloride was examined by measuring the absorbance at 562 nm.

### Angiogenesis evaluation of HUVECs

The HUVECs (3.0 × 10^4^) were seeded on the Matrigel Matrix (Corning, USA). After incubation with the sample extraction for 6 h, the vascular-like structures were stained by calcein AM and imaged using CLSM (LSM 980, Carl Zeiss, Germany). The main segment length, and number of nodes and meshes were analyzed by ImageJ.

After the cells incubated with the sample extraction and reached 100%, the cell monolayer was subject to straight scratch by a pipette. The cells were stained by calcein AM and the wound was imaged using CLSM (LSM 980, Carl Zeiss, Germany) after 0–24 h.

The migration capability of HUVECs was also analyzed using the transwell system (Corning, USA). Briefly, HUVECs (2 × 10^4^) were planted in the upper chamber, and the scaffolds of different groups were put in the lower. The migrated cells were stained with crystal violet (Leagene, China) and detected utilizing a phase-contrast microscope (Zeiss, Germany).

### Real-time quantitative polymerase chain reaction (RT-qPCR)

After osteogenic induction, total RNA was extracted from hBMSCs utilizing an RNA-Quick Purification kit (Yishan, China). The RNA was reverse transcribed using a PrimeScript RT reagent kit (Takara Biotechnology, Japan). The quantitative real-time PCR was performed with SYBR Premix Ex TaqII kit (Takara Biotechnology, Japan). The relative mRNA expression levels of early osteogenic markers (runt-related transcription factor 2 (*RUNX2*), osterix (*Osx*), *ALP*) and the late osteogenic markers (osteopontin (*OPN*) and osteocalcin (*OCN*)) were analyzed and normalized to the expression of glyceraldehyde 3-phosphate dehydrogenase (*GAPDH*). The mRNA expression levels of receptor protein-tyrosine kinase (*KDR*), CD34, Delta-like protein 4 (*DLL4*), Hes family bHLH transcription factor 1 (*HES-1*), Notch receptor 1 (*NOTCH1*), Inhibitor of DNA binding 1 (*ID-1*) and Inhibitor of DNA binding 1 (*ID-2*) were analyzed with the quantitative real-time PCR as previously mentioned. The primer sequences are shown in Table S1.

### Western blotting analysis

After osteogenic stimulation, the total protein of hBMSCs from different groups was extracted. The concentrations were evaluated using a BCA protein assay kit (Thermo Scientific, USA). Next, the protein samples were resolved by SDS-PAGE gel electrophoresis (Genscript, China), and then were transferred to polyvinylidene fluoride membranes (Millipore, USA) using a wet transfer blotting system (Bio-Rad, China). The membrane was blocked with 5% BSA at RT for 1 h. Subsequently, the membrane was incubated at 4 ℃ overnight with primary antibodies anti-RUNX2 (1:1000, Affinity, China), anti-ALP (1:1000, Affinity, China), anti-OCN (1:1000, Affinity, China), anti-OPN (1:1000, Affinity, China) and anti-GAPDH (1:1000, Affinity, China). The membrane was incubated with HRP-conjugated IgG (1:10000, Affinity, China) at RT for 1 h. The enhanced chemiluminescence solution (ECL, Millipore, USA) was used for semiquantitative analysis of the protein bands.

The protein of HUVECs from different groups was extracted and subjected to the same procedure. The primary antibodies used for incubation included anti-KDR (1:500, BIOSS, China), anti-CD34 (1:500, HUABIO, China), anti-DLL4 (1:500, BIOSS, China), anti-Itg β1 (1:500, Santa Cruz, USA), anti- integrin β3 (Itg β3) (1:1000, Abcam, USA), anti- integrin α5 (Itg α5) (1:1000, Abcam, USA), anti-vinculin (1:1000, Abcam, USA), anti-yes-associated protein 1 (YAP1) (1:1000, Proteintech, USA), anti-phospho-YAP (p-YAP) Ser127 (1:1000, Cell Signaling Technology, USA), anti- Focal adhesion kinase (FAK) (1:1000, BD, USA), anti-phospho-FAK (p-FAK) Tyr397 (1:1000, BD, USA), anti-Ras homolog family member A (RhoA) (1:1000, Cell Signaling Technology, USA), anti-Rho-associated protein kinase 1 (Rock1) (1:1000, Cell Signaling Technology, USA) and anti-GAPDH (1:1000, Affinity, China).

### Immunofluorescence (IF) staining

After osteogenic induction, the hBMSCs (2 × 10^4^) were fixed, permeabilized, blocked and incubated with anti-OCN (1:200, Affinity, China) at 4 ℃ overnight. The anti-rabbit IgG-Alexa Fluor^®^488Alexa Fluor antibody (1:200, EMAR, China) was incubated at RT for 1 h. Finally, the cells were stained with DAPI (Solarbio, China) and observed using CLSM (LSM 980, Carl Zeiss, Germany).

The HUVECs were incubated with the sample extraction from the control group or MIL-53(Fe)@AHT-1/2 group, sample conditioned medium from MIL-53(Fe)@AHT-1/2 group supplemented with both 5 mM water-soluble cholesterol (Chol, St. Louis, MO, USA) and 2 µM Latrunculin A (LatA, Calbiochem, Merck, USA) or either, respectively. The cells were incubated with primary antibodies anti-Itg β1 (1:200, Santa Cruz, USA) or anti-vinculin (1:200, Abcam, USA), and then incubated with secondary antibodies anti-mouse IgG-Alexa Fluor^®^ 594, FITC-phalloidin (Solarbio, China) and DAPI (Solarbio, China) in sequence. As for CD34 and DLL4 IF double staining, the cells were incubated with primary antibodies anti-CD34 (1:200, HUABIO, China) and anti-DLL4 (1:200, BIOSS, China) and then incubated with secondary antibodies anti-mouse IgG-Alexa Fluor^®^ 594 and anti-rabbit IgG-Alexa Fluor^®^ 488 (1:200, EMAR, China).

For cytoskeletal microfilament analysis, the HUVECs were stained with the Alexa Fluor 594-phalloidin (Solarbio, China). The length and angle of the cytoskeletal microfilaments were analyzed by ImageJ. Briefly, the F-actin fluorescent staining images were processed in grayscale for better visualization of cytoskeletal microfilaments. The length and angle of the F-actin were measured from five cells of each group, the angle of which was set to 0 -180°. The angle less than 0° was added 180° to become a positive value.

For membrane lipid raft structure detection, the cells were stained with 1 µg/mL Alexa Fluor 555 conjugated Cholera Toxin Subunit B (CT-B, Invitrogen, USA) at 4 ℃ for 10 min. The cells were detected using CLSM (LSM 980, Carl Zeiss, Germany).

### Single-cell mechanical and topographic measurements using AFM

After incubation with the sample extraction, the HUVECs were subjected to single-cell mechanical tests utilizing AFM as the pattern modified by the previous research [30, 31]. In brief, the cantilevers with 5 μm diameter spherical tips (k ~ 0.03 N/m, Novascan Technologies, Inc.) were applied for indentation tests on the perinuclear region of single cells. An approach velocity of 10 m/s was used to sample force-indentation data. The trigger forces for all samples ranged from 1.5 to 2 nN, with deflections varying from 50 to 70 nm. A modified Hertz contact model was fitted to indentation and force data in Nanoscope software (Bruker, Germany) to determine whole cell Young’s modulus. The Young’s modulus of thirty cells of each group was measured. The topography images were acquired in contact mode by means of triangular silicon nitride probes (TR400PB Asylum Research Probes, Santa Barbara) with a nominal spring constant k = 0.09 N/m, and the scanning frequency and cantilever deflection set point of 1 Hz and 1 V, respectively.

### Animals and surgical procedures

All animal experiment procedures were approved by the Institutional Animal Care and Use Committee (IACUC) of Sun Yat-Sen University (Reference: SYSU-IACUC-2022-002000). To obey the guiding principles of the three R’s, the MIL-53(Fe)@AHT-1/2 was selected for in vivo studies due to its optimal performance on angiogenic and osteogenic induction in vitro experiments. Twenty-four adult male New Zealand white rabbits (2.5–3.0 kg) were divided into three groups randomly (Blank, AHT and MIL-53(Fe)AHT-1/2) and were randomly assigned to 2 time points (4 and 12 weeks). A cylindrical critical-size bone defect (5 mm in diameter and 8 mm in height) was made in the direction perpendicular to the longitudinal axis of the tibia and the corresponding scaffold was implanted in the bone defect. After 4 weeks and 12 weeks, the tibia bones of the rabbits were harvested.

### Micro-CT analysis

The harvested tissues were scanned by micro-CT (Scanco Medical µCT 50, Switzerland) for evaluation of new bone formation surrounding the scaffold. The scanning parameters were set at 120 kV and 88 mA, with 10 μm resolution. The area of the scaffold was selected as the region of interest (ROI). The ratio of the bone volume (BV) to the total volume (TV) (BV/TV) was calculated using software (Avizo 8.1, USA).

### Histological analysis

Following micro-CT analysis, the tissues with scaffolds were fixed and dehydrated, and then embedded in polymethylmethacrylate. The embedded samples were cut into 60-µm-thick sections. Hematoxylin & eosin (H & E) staining and Goldner’s Trichrome staining were employed to evaluate the bone formation as per the manufacturer’s protocol (Servicebio, China).

The specimens used for immunohistochemistry were decalcified, dehydrated and embedding in paraffin. The slices cut from the paraffin-embedded tissues were used for subsequent experiments. The OCN or CD31 immunohistochemical assays were performed using anti-OCN (1:200, Affinity, China) or anti-CD31 (1:200, Abcam, USA) primary antibody. As for the immunofluorescence (IF) staining, the slices were incubated with primary antibodies anti-CD31 (1:200, Servicebio, China) and anti-Endomucin (Emcn) (1:200, Affinity, China) and then incubated with the secondary antibodies IgG-Alexa Fluor 594 antibody and IgG-Alexa Fluor^®^ 488 antibody (1:200, EMAR, China). The fraction area of Emcn/CD31-positive areas was quantified by ImageJ.

### Molecular docking

Molecular docking simulations are used to predict the formation of stable complexes between proteins CD34 or DLL4 with FAK, RhoA or YAP1. The aligned sequences of CD34, DLL4, FAK, RhoA and YAP1 proteins were obtained from the UniProt database. Their three-dimensional structures were predicted using AlphaFold and further refined by constructing the structures in Avogadro, optimized with the MMFF94 force field, exported in PDB format, and then imported into gauss09 for further optimization. The Hdock [32] was used to dock and score the proteins separately as the receptor and ligand. The PyMOL was used to exhibit the binding interaction geometries, with the docking affinity annotated.

### Statistical analysis

All data are expressed as the mean ± standard deviation (SD) of at least three independent experiments. Statistical analysis was performed using one-way analysis of variance (ANOVA) or Student’s t-test with GraphPad Prism 9.0 software. Differences for which *p* < 0.05 were considered statistically significant. The differences between groups or treatments were reported as ns/NS (non-significant) or significant (^*^P < 0.05, ^**^P < 0.01, ^***^P < 0.001 vs. Ctrl; ^#^*P* < 0.05, ^##^*P* < 0.01 and ^###^*P* < 0.001 among groups).

## Results

### Characterization of the scaffolds

The morphology of the scaffolds from all groups was identified at different scales. The optical images of the scaffolds used for in vitro and in vivo experiments were displayed in Fig. 2A. The SEM images of the scaffolds at low magnifications have exhibited similar surface micro-scale topography (Fig. 2B). At higher magnifications, it was observed that the surface nanotopography varied significantly. The surface of the Blank group looks smooth, while that of the AHT group was regularly distributed with nanoscale fiber-like structures with an average diameter of ∼40 nm. After modification with MIL-53(Fe) based on the AHT, MIL-53(Fe) crystals were scattered among the fiber-like structures of AHT coating. The number of MIL-53(Fe) crystals declined by degrees from MIL-53(Fe)@AHT-1 to MIL-53(Fe)@AHT-1/8 (Fig. 2C). The MIL-53(Fe) scattered among the AHT structure displayed a crystallized regular octahedron structure with an average diameter of ∼100 nm.

**Fig. 3:**
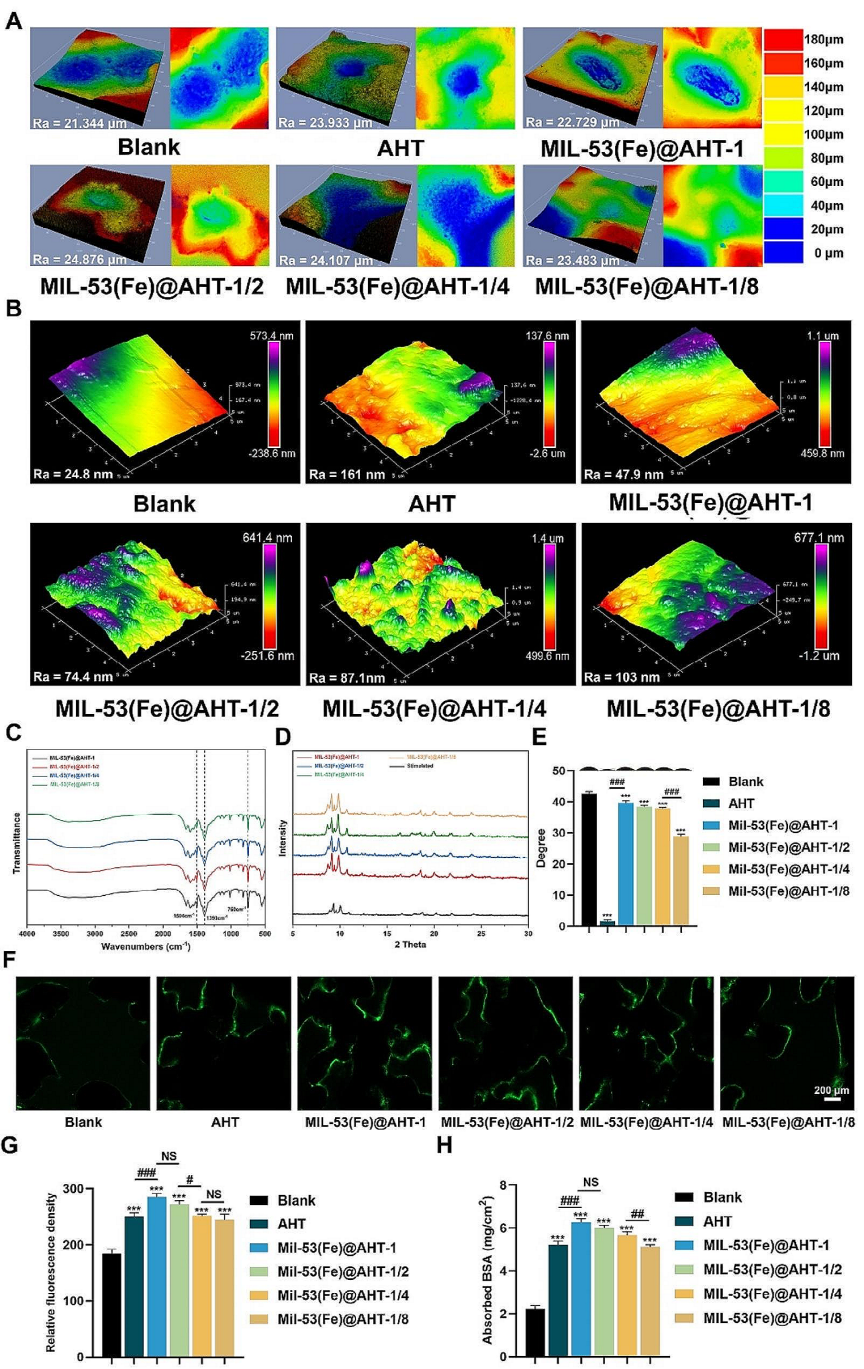
Identification of the physicochemical properties of scaffold surface. (**A**) Surface three-dimensional reconstruction view of different scaffolds by the CLSM (at microscale). (**B**) AFM analysis of the nanotopography. (**C**) FT-IR spectra of the as-prepared MIL-53(Fe) crystals. (**D**) XRD patterns of the simulated and as-prepared MIL-53(Fe) crystals. (**E**) Qualitative results of contact angles of various scaffolds. (**F**) Fluorescent images of absorption in FBS on different scaffolds and (**G**) the corresponding quantitative analysis of positive area. (**H**) Quantification analysis on protein adsorption of BSA. (*n* = 3; ^ns^*P* > 0.05, ^*^P < 0.05, and ^**^P < 0.01, ^***^P < 0.001 compared with Ctrl group; ^NS^*P* > 0.05, ^#^*P* < 0.05, and ^##^*P* < 0.01, ^###^*P* < 0.001 compared among groups)

The surface Ra and reconstruction on 3D view of the scaffolds at micro and nano scale were confirmed by confocal laser scanning microscopy (CLSM) and atomic force microscopy (AFM), respectively. The micro-scale 3D view of each scaffold was displayed in Fig. 3A. The micro-scale surface Ra of the samples from different groups was similar. The 3D view of the surface nanotopography was exhibited in Fig. 3B. The Ra at the nanoscale was significantly elevated after the AHT fabrication from 24.8 nm of the Blank group to 161 nm of the AHT group. However, the nanoscale Ra was deceased with additional MIL-53(Fe) crystals coating. As the number of MIL-53(Fe) crystals decreased, the nanoscale roughness of the surface gradually increased but was lower than that of the AHT group. Overall, there was little change in the Ra at the microscale among the groups, but the change in the nanoscale was significant. The manufacture of AHT enabled noticeable improvement on surface nanoscale roughness, but which was weakened by MIL-53(Fe) modification.

To detect on the phase purity of the as-prepared MIL-53(Fe) coating, precipitates obtained from the mother solution were dissected with FTIR and XRD spectroscopy. As for the FTIR spectra, the peak bands were at 1528, 1383 and 750 cm^− 1^ (Fig. 3C). The peak bands at 1528 cm^− 1^ and 1383 cm^− 1^ were attributed to the asymmetric and symmetric vibrational modes of the C-O bond in the -COOH group, respectively, while the peaks at 750 cm^− 1^ indicated the bending vibration mode of the C-H bond of the benzene ring, revealing that the crystals were of pure phase [29, 33]. The results of the XRD pattern agreed well with the simulated patterns [29] (Fig. 3D), implying that successful synthesis of MIL-53(Fe) coating has been achieved on the substrates.

The wettability of different scaffolds was analyzed by the contact angle measurement. As shown in Fig. 3E, the contact angle for the scaffold of the Blank group was approximately 42.2°, while that for the AHT group was relatively small (< 3°). Furthermore, the contact angle declined gradually from MIL-53(Fe)@AHT-1 to MIL-53(Fe)@AHT-1/8 corresponding to the decreased inclination of the crystal number, which was similar to that of other titanium stents modified with MOF nanoparticles [19, 34].

The quantified analysis on the fluorescent intensity of FITC-FBS absorbed in the scaffolds and Bovine serum albumin (BSA) absorption reflected the protein adsorption ability. The results of protein adsorption experiments demonstrated that either for FBS (Fig. 3F-G) or for BSA (Fig. 3H), the AHT and MIL-53(Fe) modification markedly elevated the degree of protein adsorption. The MIL-53(Fe)@AHT-1 and MIL-53(Fe)@AHT-1/2 groups performed better in protein adsorption.

### Cytotoxicity assessment of the scaffolds

**Fig. 4:**
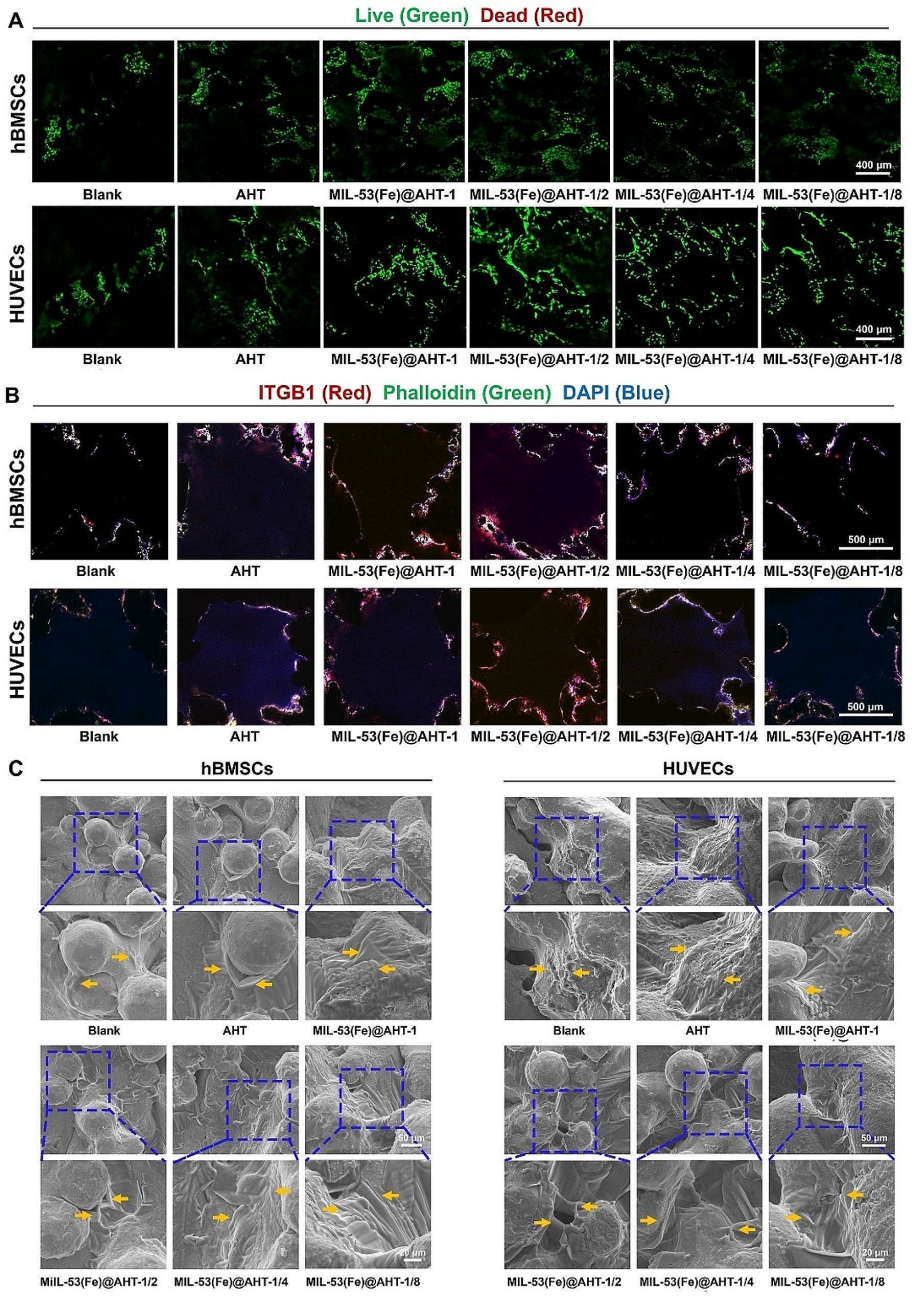
Cytotoxicity assessment of the scaffold. (**A**) The live-dead images of hBMSCs and HUVECs cultured on different substrates. Live: green; Dead: read. (**B**) Representative fluorescent images of hBMSCs and HUVECs seeded on the scaffolds for 24 h. (**C**) The SEM images of hBMSCs and HUVECs attached to the scaffolds. (*n* = 3; ^ns^*P* > 0.05, ^*^P < 0.05, and ^**^P < 0.01, ^***^P < 0.001 compared with Ctrl group; ^NS^*P* > 0.05, ^#^*P* < 0.05, and ^##^*P* < 0.01, ^###^*P* < 0.001 compared among groups)

To observe the cell viability incubated on the scaffolds, hBMSCs and HUVECs were subject to the live/dead staining. The hBMSCs and HUVECs (Fig. 4A) seeded on the scaffolds for 3 days exhibited high viability and dead cells were hardly detected. Additionally, the number of cells cultivated in samples with MIL-53(Fe)@AHT coating far surpassed that in the Blank and AHT groups. The survival and proliferation of both cells analyzed by the CCK-8 kit indicated a similar tendency (Fig. S1A-B). The results evaluated at the duration of 1, 3, 5 and 7 d showed that all groups showed higher Optical density (OD) values over time, indicating that the samples exhibited better cytocompatibility. From 5 days onwards, the OD value for the scaffolds with MIL-53(Fe)@AHT coating was markedly higher than that of the AHT and Blank groups, revealing that the MIL-53(Fe) coating was beneficial for enhancing of cell viability. In particular, the cells in the MIL-53(Fe)@AHT-1/2 group presented the optimal viability.

To analyze the cell attachment on the substrates, the Itg β1 expression of cells was evaluated by IF staining after 24-hour incubation. Meanwhile, the cell skeleton and nucleus were also stained to further reflect the cell spreading. The IF staining exhibited that the adhesion and distribution of hBMSCs and HUVECs on the scaffold presented an analogous tendency (Fig. 4B). The cells seeded on the scaffolds for 24 h spread well and mainly distributed along the pores. In particular, more cells were discovered attached and distributed in the MIL-53(Fe)@AHT-1/2 group than in other groups. The IF staining images of Itg β1 and semi-quantification analysis on Itg β1 expression of hBMSCs (Fig. S1C-D) and HUVECs (Fig. S1E-F) on the substrates also confirmed the cell adhesion properties of each scaffold. It was identified that the samples with MIL-53(Fe)@AHT coating contributed to cell adhesion, indicating that the constructed coating performed favorable biocompatibility. Additionally, an improvement in the adhesion properties affected by MIL-53(Fe)@AHT fabrication was more significant in HUVECs than in hBMSCs.

The cell morphology on different scaffolds incubated for 3 days was also observed using SEM to reflect the degree of cell adhesion. As shown in Fig. 4C, although abundant filopodia were found on the leading edge of the hBMSCs seeded on the scaffolds of the Blank group, the spreading area of the cell was limited and small amount cells aggregated together. As for the AHT groups, more hBMSCs were attached to the surface as well as the cell spreading out and initiating clustering. The construction of MIL-53(Fe) coating further facilitated cell stretching and connecting into clusters of the hBMSCs. In terms of HUVECs, globular cells with limited spreading were observed, and the cells were arranged in a beaded pattern in the Blank group. The HUVECs seeded on the scaffolds of the AHT group were arranged in strips, indicating the cell stretch area increases. Furthermore, clusters of cells cover large areas in the substrates with MIL-53(Fe)@AHT coating. The hBMSCs and HUVECs in the scaffolds with MIL-53(Fe)@AHT coating exhibited polygonal morphology with plentiful flat lamellipodia. In conclusion, the MIL-53(Fe)@AHT coating was beneficial to cell spreading and connection, thus possessing preferable cytocompatibility.

### Stimulation of osteogenic differentiation of hBMSCs in vitro by the scaffolds

**Fig. 5:**
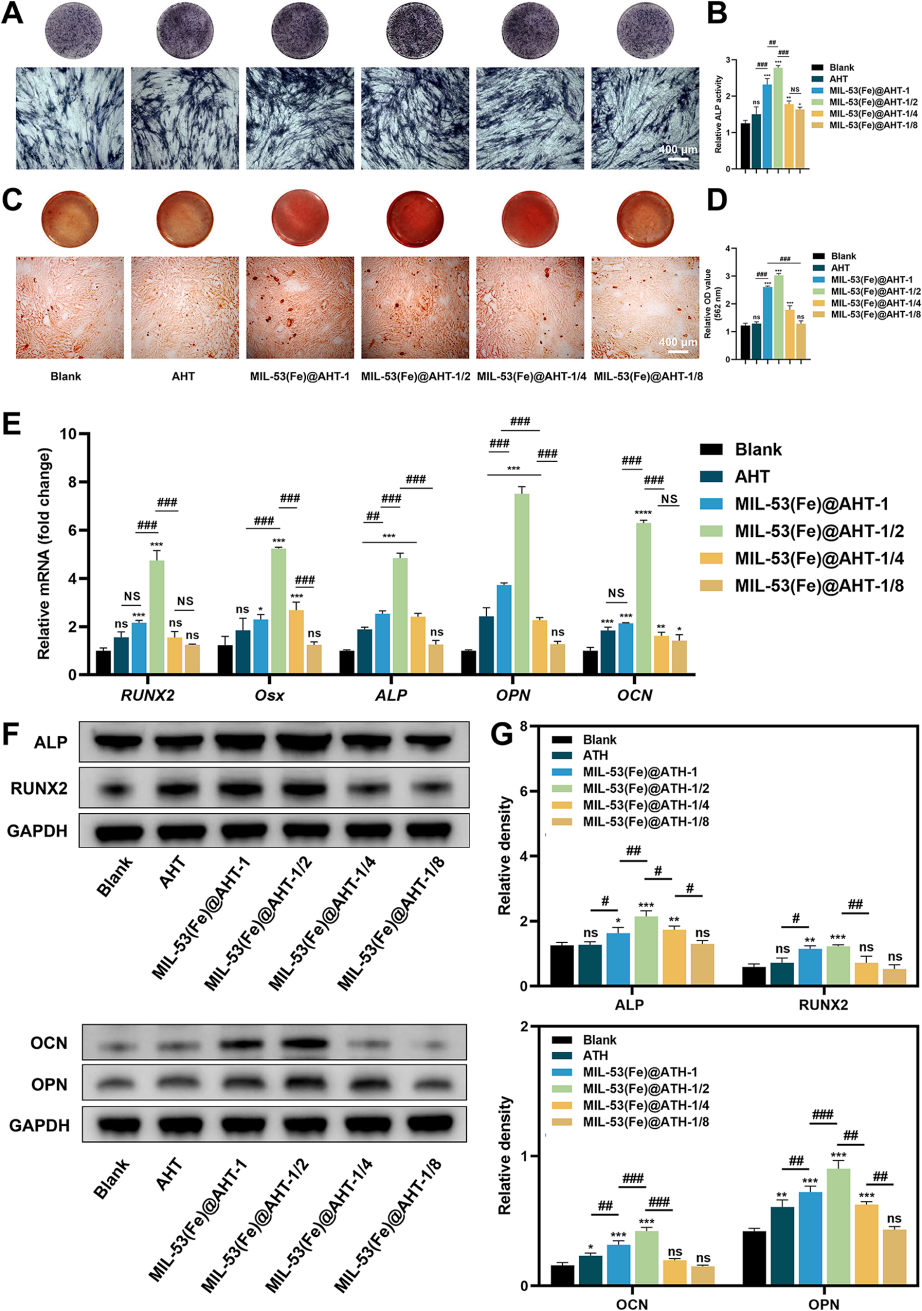
In vitro detection of osteogenic differentiation of hBMSCs. (**A**) The gross images (upper) and microscopic images (lower) with ALP staining and (**B**) ALP activity cultured in conditioned medium from different samples. (**C**) The gross images (upper) and microscopic images (lower) with Alizarin red S staining of mineralized nodules and (**D**) corresponding semi-quantified analysis of hBMSCs induced by different scaffolds. (**E**) RT-qPCR analysis on the expression of the early osteogenic markers (*RUNX2*,* Osx* and *ALP*) and the late osteogenic markers *(OPN* and *OCN)* of hBMSCs incubated on different scaffolds. (**F**) Western blotting images of RUNX2, ALP, OCN, OPN and GAPDH of hBMSCs seeded on different substrates. (**G**) The quantified protein levels of RUNX2, ALP, OCN and OPN. (*n* = 3; ^ns^*P* > 0.05, ^*^P < 0.05, and ^**^P < 0.01, ^***^P < 0.001 compared with Ctrl group; ^NS^*P* > 0.05, ^#^*P* < 0.05, and ^##^*P* < 0.01, ^###^*P* < 0.001 compared among groups)

To evaluate the osteogenic differentiation of hBMSCs induced by the samples, the ALP staining assays and the relative ALP activity were conducted to verify the early stage of osteogenesis, while the ARS staining assays were performed to investigate the late osteogenic stage. The results of the ALP staining assay and ALP activity quantification indicated that as compared to the Blank group, hBMSCs cultivated with MIL-53(Fe)@AHT coating had more ALP-positive areas and intense ALP staining, while the cells cultivated in the MIL-53(Fe)@AHT-1/2 group presented the best ALP activity (Fig. 5A-B). In terms of the ARS staining, the hBMSCs in MIL-53(Fe)-1, MIL-53(Fe)-1/2 and MIL-53(Fe)-1/4 group had 1.5-fold higher staining than the Blank group. More matrix mineralization was observed in the MIL-53(Fe)-1/2 group than in the other MIL-53(Fe) modification groups (Fig. 5C), which was affirmed by the semiquantitative analysis of the mineralized nodules as well (Fig. 5D). In addition, the relative expression of osteogenic-related genes (***RUNX2***, ***Osx***, ***ALP***, ***OPN*** and ***OCN***) were analyzed utilizing RT-qPCR. The relative expression of early osteogenic markers, *RUNX2*,* Osx* and *ALP*, were markedly upregulated after 7 days in hBMSCs of MIL-53(Fe)@AHT-1/2 group, whereas the significant upregulation expression of late osteogenic markers, *OPN* and *OCN*, was detected after 14-day incubation (Fig. 5E). Furthermore, the protein expression of ALP and RUNX2 in hBMSCs of different groups was analyzed after 7-day induction, and the protein expression of OCN and OPN was analyzed after 14-day induction. The results of western blotting revealed that the relative protein expression of RUNX2, ALP, OCN and OPN were markedly increased in the MIL-53(Fe)@AHT-1/2 group in comparison with the other groups (Fig. 5F). The quantified analysis also verified the obtained consequences (Fig. 5G). The IF staining was further performed to evaluate the expression of RUNX2 and OPN. As expected, it presented a similar expression pattern as the results of RT-qPCR and Western blotting mentioned earlier (Fig. S2). The hBMSCs in the MIL-53(Fe)@AHT-1/2 group possessed the most expression of RUNX2 and OPN.

### Activation of endothelial tip cells and enhancement of angiogenesis in vitro by the scaffolds

**Fig. 6:**
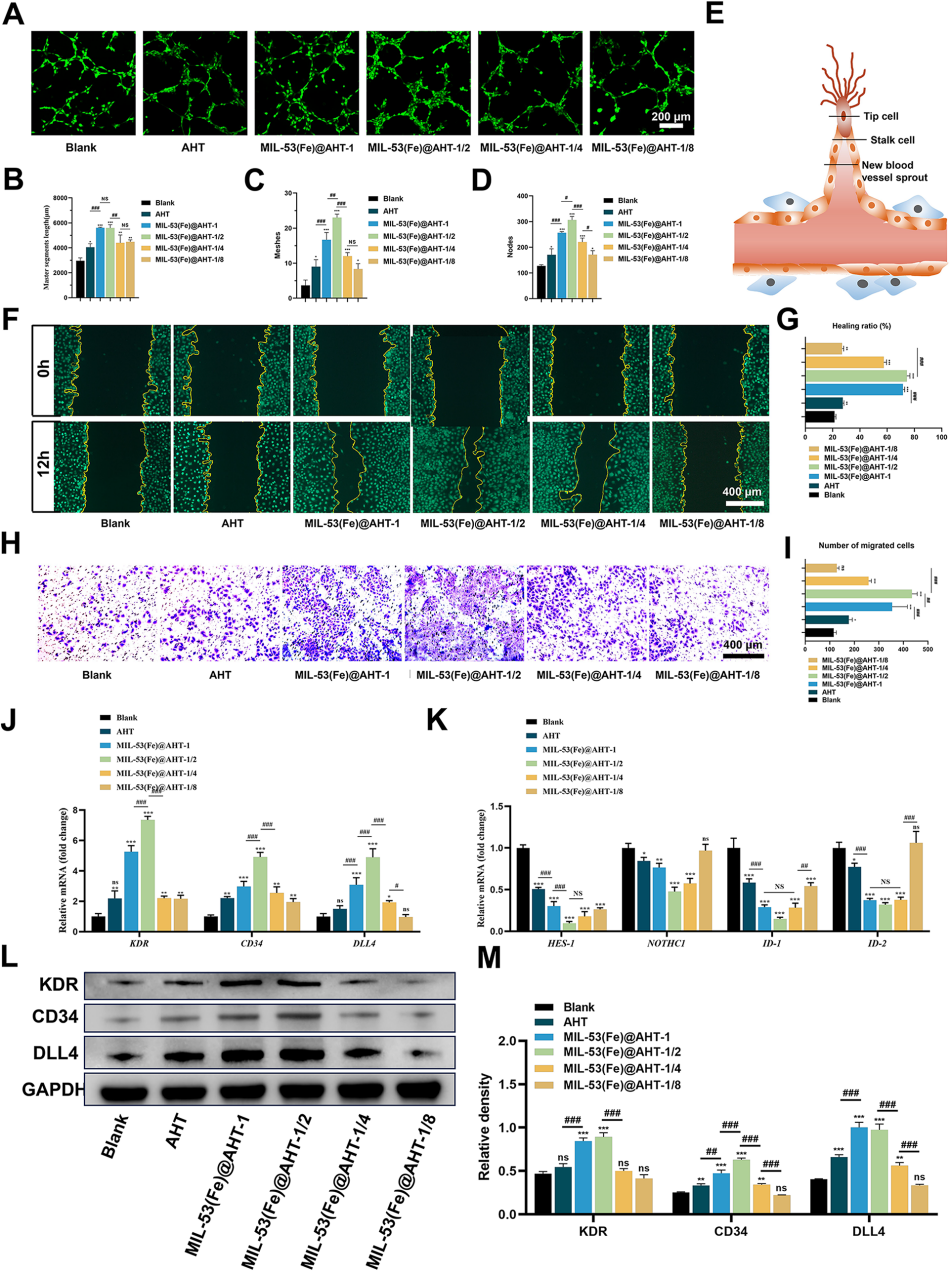
The stimulation of angiogenesis and activation on tip cell differentiation of HUVECs. (**A**) Representative images of the tube formation and quantified analysis on (**B**) master segments length, (**C**) meshes and (**D**) nodes of HUVECs cultivated in different sample conditioned medium for 6 h. (**E**) Schematic of selection on endothelial tip-stalk cell in sprout vessels during angiogenesis. (**F**) Wound healing test in HUVECs cultured in different sample conditioned medium. (**G**) The quantified analysis on wound healing area (%). (**H**) The microscopic images and (**I**) the quantified numbers of the migrated HUVECs treated with different sample conditioned mediums incorporated in transwell system for 24 h. (**J**) The mRNA expression of tip-cell-associated and stalk-cell-associated genes (**K**) in HUVECs cultured in different sample conditioned medium for 24 h. (**L**) Immunoblotted images for KDR, CD34, DLL4 and GAPDH of HUVECs induced by the scaffolds from diverse groups. (**M**) Analyses of blots showing the values for KDR, CD34 and DLL4. (*n* = 3–5; ^ns^*P* > 0.05, ^*^P < 0.05, and ^**^P < 0.01, ^***^P < 0.001 compared with Ctrl group; ^NS^*P* > 0.05, ^#^*P* < 0.05, and ^##^*P* < 0.01, ^###^*P* < 0.001 compared among groups)

The impact of the different samples on facilitation in angiogenesis and activation of tip cells was further explored. The tube formation assay of HUVECs in different samples shown in Fig. 6A indicated that the groups with MIL-53(Fe)@AHT coating exhibited a superior tubular morphology compared with the Blank group. The master segment length (Fig. 6B), tube meshes (Fig. 6C) and number of nodes (Fig. 6D) of HUVECs in substrates with MIL-53(Fe)@AHT coating were significantly increased compared with the Blank group. Particularly, these parameters were over two-fold greater in the MIL-53(Fe)@AHT-1/2 than those in the Blank group. The endothelial tip-stalk cell selection is vital to angiogenesis [4, 35]. After activation of the tip cell, the adjacent stalk cells will be facilitated to proliferate and elongate, thus inducing vascular lumen formation (Fig. 6E). Therefore, activation of the tip cell induced by different samples was investigated.

The endothelial tip cells were characterized by powerful migration ability. The migration of HUVECs in different samples was evaluated by wound healing assay. It was indicated that the wound area in the MIL-53(Fe)@AHT groups was visibly reduced after 24 h incubation. Especially the wound area in MIL-53(Fe)@AHT-1/2 was the smallest (Fig. 6F). The quantitative analysis of the wound healing ratio (Fig. 6G) was in line with the observation. The transwell system was employed to further investigate the migration ability of HUVECs in various groups. The results of the observation (Fig. 6H) and quantification (Fig. 6I) of the migrated cells also indicated that the samples with MIL-53(Fe)@AHT coating improve the migration ability of HUVECs in comparison with the Blank group, in particular the MIL-53(Fe) @AHT-1/2 group. Furthermore, the relative levels of tip cell-related genes *KDR*, *CD34* and *DLL4* and stalk cell-related genes *HES-1*, *NOTHC1*, *ID-1* and *ID-2* were assessed using RT-qPCR. The tip cell-related genes were significantly upregulated (Fig. 6J) in HUVECs of MIL-53(Fe)@AHT groups after 24 h incubation. Specifically, the expression of *KDR*,* CD34 and DLL4* of the HUVECs in the MIL-53(Fe)@AHT-1/2 group were increased about 5-, 3- and 3-fold, respectively, compared to the Blank group. Additionally, the relative level of stalk-cell-genes expression was shown the opposite trend (Fig. 6K). The relative protein expression of tip cell-related genes was further investigated. The western blotting revealed that the relative expression of KDR, CD34 and DLL4 were markedly upregulated in the samples with MIL-53(Fe)@AHT coating compared with the Blank group (Fig. 6L). The quantified analysis on the basis of the band density also verified the acquired results (Fig. 6M). Collectively, the MIL-53(Fe)@AHT coating had access to encouraging the activation of endothelial tip cells and promoting angiogenesis, especially the MIL-53(Fe)@AHT-1/2 group.

### Enhanced vascularized bone regeneration in vivo by the scaffolds

**Fig. 7:**
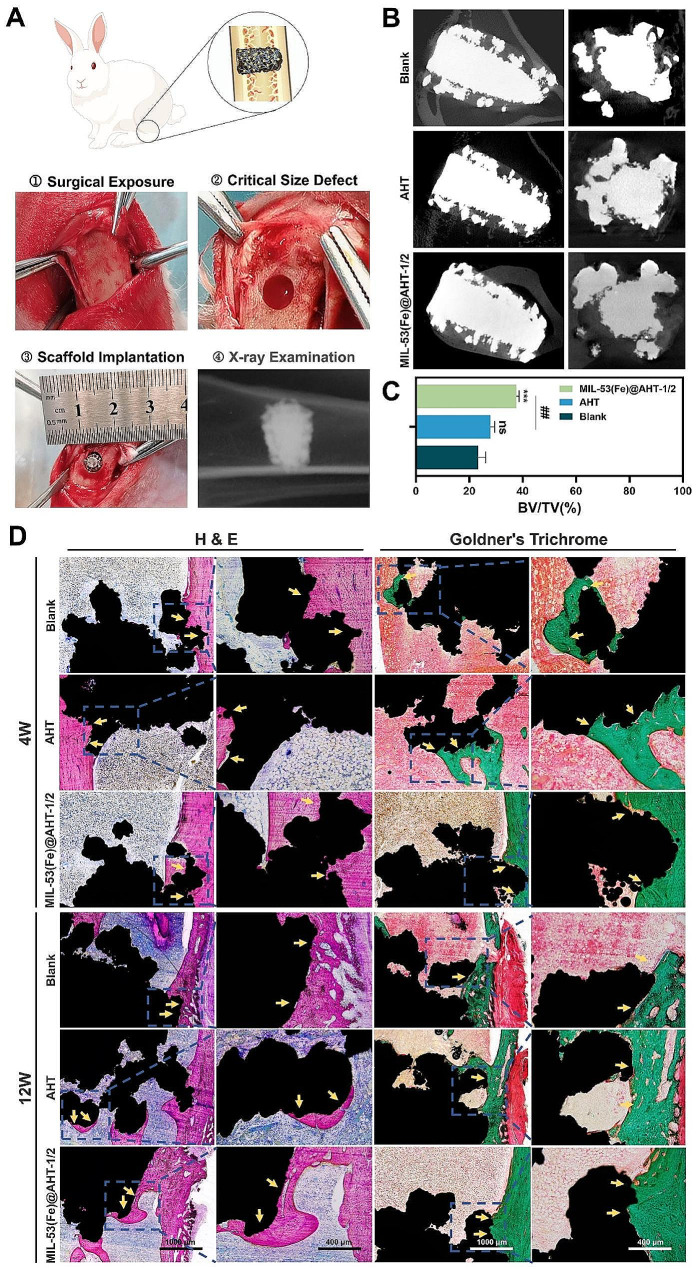
Implantation of scaffolds and evaluation of the regenerated bone with micro-CT and histological analysis. (**A**) The surgical process of sample implantation in rabbits with critical tibial defects. (**B**) Micro-CT reconstruction of bone regeneration with the scaffolds 12-weeks post-surgery. (**C**) Analysis on volume/total volume (BV/TV) of the bone defect 12 weeks after implantation surgery. (**D**) Representative images of H & E staining and Goldner’s Trichrome staining of newly formed bone at 4- and 12-weeks post-operation. The black part indicates the scaffolds and the yellow arrows indicate the newly formed bone. (*n* = 3; ^NS^*P* > 0.05, ^*^P < 0.05, and ^**^P < 0.01, ^***^P < 0.001 compared with Ctrl group; ^ns^*P* > 0.05, ^#^*P* < 0.05, and ^##^*P* < 0.01, ^###^*P* < 0.001 compared among groups)

The vascularized bone regeneration abilities of the samples were evaluated utilizing the critical-sized tibia bone defect models in rabbits. Twenty-four adult male New Zealand white rabbits (2.5–3.0 kg per rabbit) were randomly divided into three groups (Blank, AHT and MIL-53(Fe)AHT-1/2) and were randomly assigned to 2 time points (4 and 12 weeks). After the critical-size bone defects were constructed, the scaffolds were implanted and were initially evaluated by the X-ray examination (Fig. 7A). At 12 weeks post-operation, more volume of newly growing bone was discovered elevated in the MIL-53(Fe)@AHT-1/2 group. The new bone tissues were found to be ingrowth into the scaffolds (Fig. 7B). A significantly higher BV/ TV value was found in the MIL-53(Fe)@AHT-1/2 groups (37.60 ± 1.0%) compared with that in the AHT group (27.88 ± 1.64%, *p* < 0.05) and Blank group (23.28 ± 2.80%, *p* < 0.05) (Fig. 7C).

The H & E and Goldner’s Trichrome staining assay further determined the regenerated bone formation among three groups. In terms of H & E staining (Fig. 7D), at 4 weeks after surgery, more regenerated bone was found ingrowth of the scaffolds of the MIL-53(Fe)@AHT-1/2 group than the other groups. At 12 weeks postoperatively, the new bone tissue of all three groups presented increased ingrowth of the scaffolds, while the MIL-53(Fe)@AHT-1/2 performed the best. The histomorphometry analysis also revealed a much higher bone volume in MIL-53(Fe)@AHT group at 4 and 12 weeks compared with the AHT group and Blank group (Fig. S3A). Additionally, no apparent connective tissue hyperplasia or poor bone healing was observed in the H & E staining slices, indicating the good biocompatibility of the scaffold. Similar to the H & E staining, Goldner’s Trichrome staining indicated that more consecutive collagen fiber bundles and ossified tissues were arranged compactly in the MIL-53(Fe)@AHT group compared with the other groups (Fig. 7D). The histomorphometry analysis also confirmed the increased new bone formation in MIL-53(Fe)@AHT-1/2 in comparison with the other groups (Fig. S3B).

**Fig. 8:**
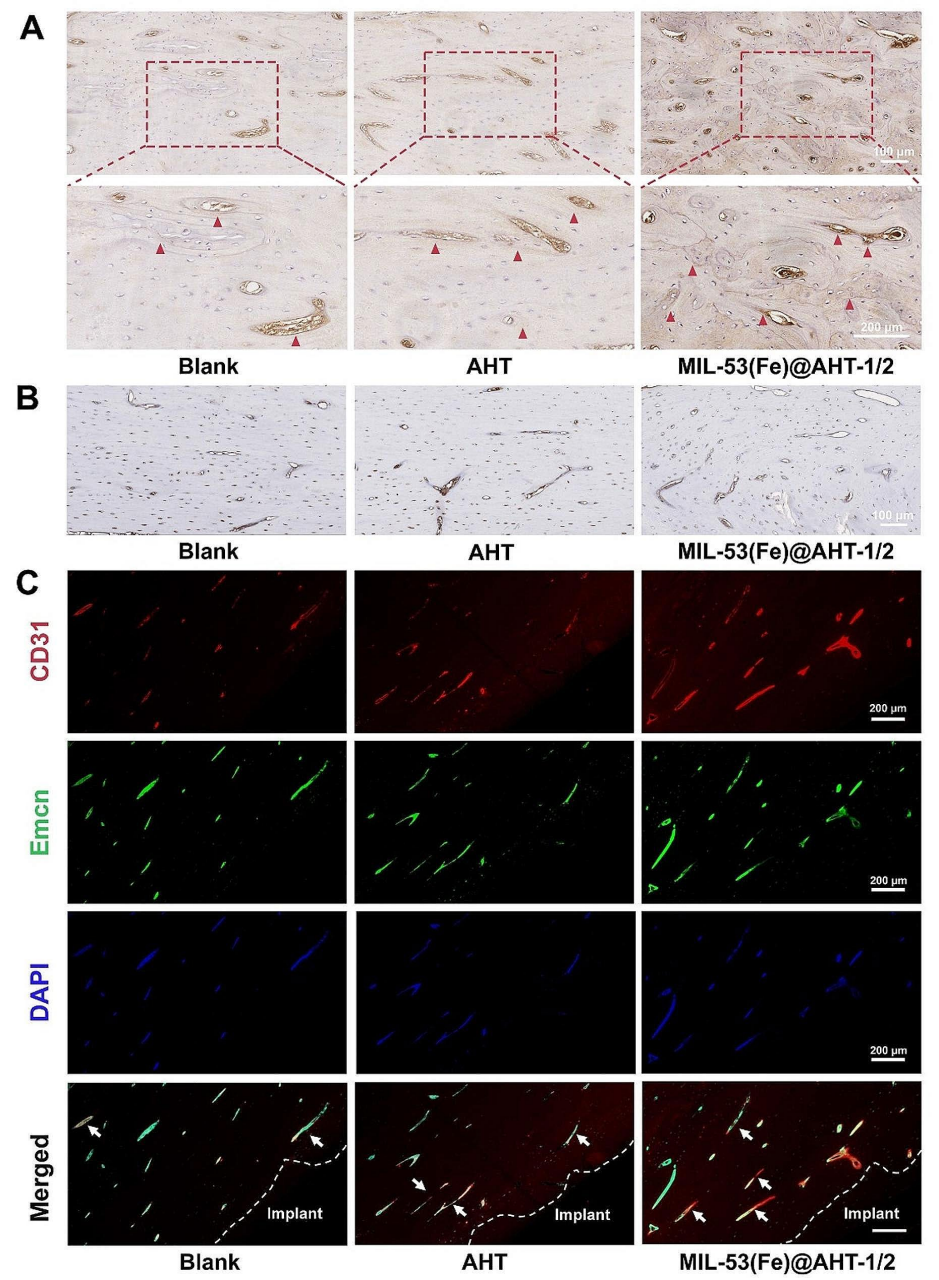
Immunohistochemical and IF staining on vascularized bone regeneration after 12 weeks. (**A**) The immunohistochemical staining images of OPN. The red arrows indicate the positive area. (**B**) The immunohistochemical staining images of CD31. (**C**) The IF staining images of CD31/Emcn positive area. The white arrows indicate the positive area

Enhanced vascularized bone regeneration in vivo was also evaluated by the expression of a late marker of the osteogenic differentiation, ***OCN***, and a well-defined marker of the angiogenesis, ***CD31***, in immunohistochemical staining 12 weeks after surgery. The immunohistochemical staining of OCN revealed that the expression of OCN presented in MIL-53(Fe)@AHT-1/2 group was superior to the other groups (Fig. 8A). The quantified analysis of the positive areas revealed the highest OCN expression in the MIL-53(Fe)@AHT-1/2 group as well (Fig. S3C). The results of CD31 expression detected by immunohistochemical staining indicated that MIL-53(Fe)@AHT-1/2 group has significantly more number and higher density of neo-vessels than the other groups (Fig. 8B), which was also confirmed by the quantitative analysis on positive staining area of CD31 (Fig. S3D). Interestingly, combined analysis on the OCN-positive and CD31-positive areas, some OCN expressed cells were found located directly adjacent to the CD31 positive cells. Furthermore, the formation of H-type blood vessels (*CD31*^high^*Emcn*^high^) was evaluated by dual IF staining. It was indicated that compared with the other groups, the CD31/Emcn positive staining area was significantly increased in the MIL-53(Fe)@AHT-1/2 group (Fig. 8C). The quantitative analysis on the fraction of type H vessels areas was in accordance with the observation (Fig. S3E). Therefore, the scaffolds of MIL-53(Fe)@AHT group were proved for enhancement on vascularized bone regeneration of the critical-size bone defect.

### Facilitating ECM protein adsorption and mechanotransduction process of HUVECs by the scaffolds

**Fig. 9:**
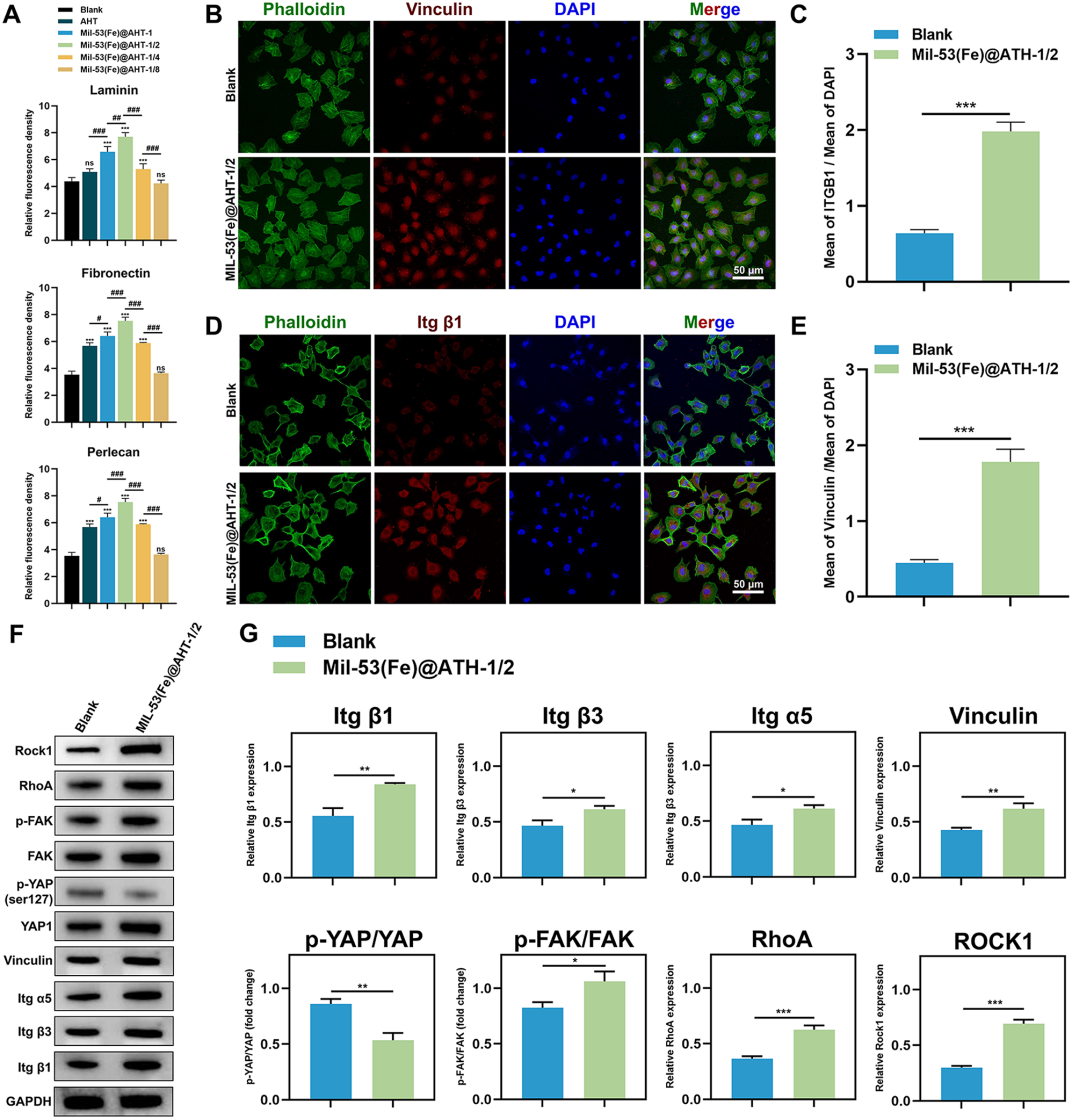
Effect on ECM proteins adsorption and mechanotransduction of HUVECs by the scaffold. (**A**) Quantification analysis of fluorescent staining on protein adsorption of laminin, fibronectin and perlecan. (**B**) Immunofluorescent images for vinculin expression of HUVECs and (**C**) corresponding quantification analysis. (**D**) Immunofluorescent images for Itg β1 expression of HUVECs and (**E**) corresponding quantification analysis. (**F**) Immunoblotted images for mechanotransduction-related gene expression of HUVECs induced by the scaffolds from diverse groups and (**G**) the corresponding quantified analysis on protein levels. (*n* = 3; ^ns^*P* > 0.05, ^*^P < 0.05, and ^**^P < 0.01, ^***^P < 0.001 compared with Ctrl group; ^NS^*P* > 0.05, ^#^*P* < 0.05, and ^##^*P*#x2009;< 0.01, ^###^*P* < 0.001 compared among groups)

The mechanism underlying the MIL-53(Fe)@AHT modified scaffolds mediated improving angiogenesis was further investigated. MIL-53(Fe)@AHT-1/2 group was chosen for the subsequent experiments because it presented the optimal potency for promoting angiogenesis. Mechanotransduction plays a significant role in the regulation of angiogenesis [36]. The adsorption ability of several common ECM proteins associated with mechanotransduction process such as laminin, fibronectin and perlecan was evaluated in IF staining assay. The results of fluorescent images (Fig. S4) and corresponding quantified analysis on the fluorescent intensity (Fig. 9A) reflected that the MIL-53(Fe)@AHT-1/2 group presented optimal adsorption capability of these proteins. The expression of adhesion biomolecules initiating the mechanotransduction process was assessed. The increased expressions of Itg β1 and vinculin of HUVECs in MIL-53(Fe)@AHT group were confirmed in the IF staining assay (Fig. 9B-E) and western blotting (Fig. 9F-G). The improved expression of other mechanosensing molecules including Itg β3 and Itg α5 were also verified by western blotting (Fig. 9F-G). The expression of signaling pathways adjusting intracellular mechanotransduction was also detected by western blotting. The phosphorylation level of FAK and expression level of RhoA and Rock1 were elevated by 50%, while the phosphorylation level of YAP was decreased by 50% in HUVECs of MIL-53(Fe)@AHT-1/2 group (Fig. 9F-G). Hence, the MIL-53(Fe)@AHT coating could enhance the mechanotransduction process of HUVECs through promoting ECM protein adsorption. The results of molecular docking in Fig. S5 indicated that all binding energies were negative. According to the docking scores, RhoA had the strongest binding affinity for protein CD34 or DLL4, which indicated the *RhoA*/*ROCK* pathway may serve as a crucial role in it.

### Effect of manipulating cell stiffness on the activation of tip cell and promoting angiogenesis by the scaffolds

**Fig. 10:**
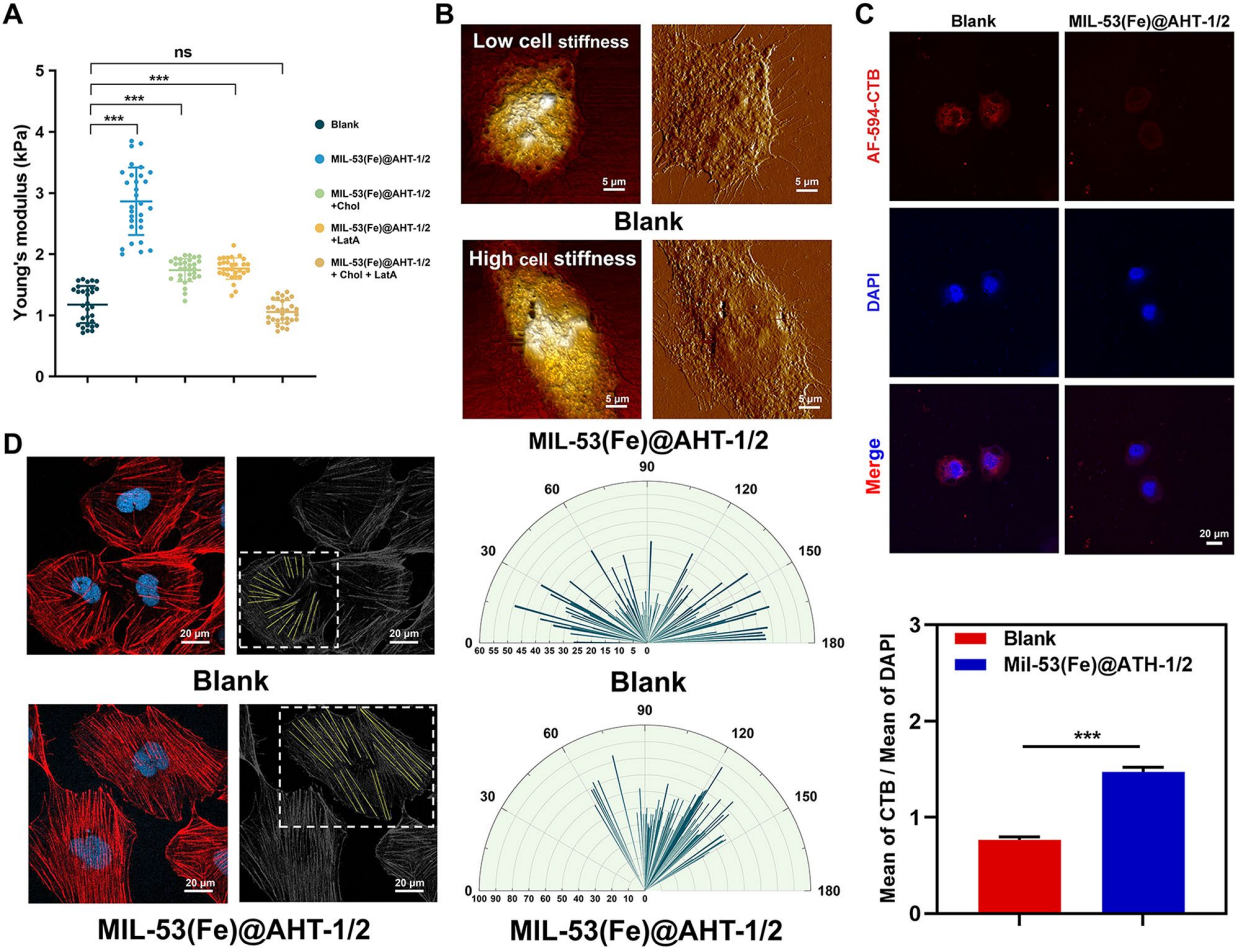
Cell stiffness evaluation of HUVECs co-cultured with the scaffolds. (**A**) Relative cell cortical stiffness of HUVECs in Blank group and MIL-53(Fe)@AHT-1/2 group, the sample conditioned medium supplemented with either water-soluble cholesterol or LatA or both, respectively (MIL-53(Fe)@AHT-1/2 + Chol, MIL-53(Fe)@AHT-1/2 + LatA and MIL-53(Fe)@AHT-1/2 + Chol + LatA) determined by AFM (*n* = 30). (**B**) Topographical images of HUVECs taken with the AFM. (**C**) Membrane lipid raft structure of HUVECs cultured with sample conditioned medium from Blank group and MIL-53(Fe)@AHT-1/2 group and the corresponding quantitative analysis of CTB (*n* = 3). (**D**) Fluorescent staining and identification of F-actin in HUVECs and the corresponding analysis of the length and angle of F-actin in HUVECs by ImageJ (*n* = 5). (^ns^*P* > 0.05, ^*^P < 0.05, and ^**^P < 0.01, ^***^P < 0.001 compared with Ctrl group; ^NS^*P* > 0.05, ^#^*P* < 0.05, and ^##^*P* < 0.01, ^###^*P* < 0.001 compared among groups)

Cell stiffness was mainly decided by membrane cholesterol and the underlying actin cortex [16, 17, 37]. Decreased membrane cholesterol content or uniform orientation of actin cortex is in accordance with increased cell stiffness. The cell stiffness of HUVECs in the Blank group and MIL-53(Fe)@AHT-1/2 group was measured by AFM. The results revealed that the Young’s modulus of HUVECs in the MIL-53(Fe)@AHT-1/2 group was about triple that of cells in the Blank group (Fig. 10A). The topographical images of the HUVECs in Blank group and MIL-53(Fe)@AHT-1/2 group were taken by AFM (Fig. 10B). The correspondence between the cell topology and cell stiffness was consistent with the researches previously reported [30]. The aspect ratio defined as the width (the smallest diameter of the cell)/length (the largest diameter of the cell) of the stiffer cell was reduced. In addition, the HUVECs in the MIL-53(Fe) group were characterized by more and longer pseudopodia than the Blank group. From the perspective of the cholesterol content of the plasm membrane, immunofluorescent staining of lipid rafts (cholesterol-enriched membrane microdomains [38]) indicated that the membrane cholesterol content of HUVECs in the MIL-53(Fe)@AHT-1/2 was distinctly decreased (Fig. 10C), which reflected that the cell stiffness was elevated. It was further verified by the results that the elevated cell stiffness of HUVECs induced by MIL-53(Fe)@AHT-1/2 groups was partially rescued by supplement of Cholesterol (Chol) (Fig. 10A). Furthermore, fluorescent staining and identification of F-actin depicted that the F-actin microfilaments of HUVECs in MIL-53(Fe)@AHT-1/2 group were more oriented and denser (Fig. 10D). The further analysis on length and angle of the F-actin microfilaments extracted from the HUVECs in both groups also confirmed the analogous tendency (Fig. 10D). The polymerization of F-actin microfilaments was also another critical feature of stiffer cells. The increased cell stiffness of the HUVECs induced by MIL-53(Fe)@AHT-1/2 was identified to be weakened by treatment with LatA (a reagent to depolymerize actin filaments [39]), and was completely rescued by co-incubation with both Chol and LatA (Fig. 10A). Hence, it can be concluded that the cell stiffness of HUVECs could be increased by the samples with MIL-53(Fe)@AHT coating.

The role of cell stiffness in the activation of tip cells and angiogenesis by the MIL-53(Fe)@AHT-1/2 group was further explored. The dual IF staining for CD34 and DLL4 (Fig. S6A) and quantified analysis on the percentage of positive CD34/ DLL4 cells (Fig. S6B) indicated that the co-localization coefficient of CD34 and DLL4 was increased in MIL-53(Fe)@AHT-1/2 groups, which was partially reduced by co-incubation with Chol or LatA and was completely rescued by supplement with both of them. The expression of tip cell-related genes detected by western blotting also confirmed the same tendency (Fig.S6C-D). The tube formation assay verified that the superior degree of tube formation in MIL-53(Fe) was partially lessened by treatment with cholesterol or LatA and entirely recovered by supplementing with both of them (Fig. S7A). The quantified analysis of the master segment length (Fig. S7B), tube meshes (Fig. S7C) and numbers of nodes (Fig. S7D) was also in accordance with the observation. The migration efficiency of the HUVECs evaluated by the wound healing assay (Fig. S7E-F) and the transwell system (Fig. S7G-H) also presented a similar tendency. In conclusion, the MIL-53(Fe)@AHT group facilitated the activation of tip cells and angiogenesis by enhancement of the cell stiffness of HUVECs.

## Discussion

Over the years, porous titanium scaffolds, manufactured for reduced stress shielding effect and stimulated new bone and vessel ingrowth, received considerable attention in the development of substitutes for bone repair [40]. Suitable pore sizes of bone repair scaffolds not only enabled sufficient vascularization of the material and prevented hypoxic conditions in the inner regions but also adjusted the mechanical properties of the scaffolds to be compatible with the human bone tissue with the purpose of maintaining the support strength and reducing the occurrence of stress-shielding [6, 40]. A 3D-printed Ti-6Al-4 V scaffold with larger porosity and irregularly designed pore size of 1000 μm was manufactured in our previous studies, which was characterized with the appropriate elastic modulus of 15.53 ± 0.55 GPa closest to that of human bone tissue (4 GPa to 20 GPa) and facilitated the ingrowth and interconnectivity of newly formed vessels and bone tissue [6]. Hence, the scaffold was a promising substrate for further improvement to achieve superior efficacy of large bone defect reconstruction. However, the bioinert surface of titanium, adverse to sufficient vascularization, was unfavorable to the survival of the scaffold [7]. In addition, the activation of endothelial tip cells, the angiogenic stage contributing to accelerated vascularization, was increasingly focused in bone tissue engineering recently [4]. Therefore, on the basis of porous bionic 3D-printed Ti-6Al-4 V scaffold, this study proposed a biofunctionalization strategy forming composite MIL-53(Fe)@AHT coating to promote sufficient vascularization in bone regeneration and further explored its possible mechanism to facilitate activation of endothelial tip cell and angiogenesis.

The bone reconstruction scaffolds fabricated by nanoscale MOFs, such as ZIF-8 [19], Mg/Zn-MOF74 [41], bio-MOF [34, 42], etc., have presented excellent biocompatibility. The 3D-printed porous Ti-6Al-4 V scaffolds with MIL-53(Fe)@AHT coating constructed in this study also had favorable biocompatibility (Fig. 4 & S1). Integrin act as a vital role in the cell-ECM adhesion [43]. The expression of *Itg β1* was significantly upregulated in hBMSCs and HUVECs incubated in the scaffolds with MIL-53(Fe) modification (Fig. S1D&F), indicating the anchoring between the cells and scaffolds was markedly facilitated, which was attributed to its superior protein absorption ability (Fig. 3G-H). It was identified that the titanium scaffold modified with MOFs also possesses favorable osteoinductivity [19, 22, 34, 41, 42, 44]. ZIF-8, the most commonly used MOF material to improve the bioactivity of the substrates, was reported to promote osseointegration at the bone-implant interface [19]. Fabrication of Mg/Zn-MOF74 on titanium implants reported by Shen et al. was characterized by antibacterial, anti-inflammatory and pro-osteogenic properties at infected bone positions [41]. Mg-MOF-74 loaded with icariin was wrapped in titanium scaffold for pro-osteoblastogenesis by controlling the release of icariin and Mg^2+^ [22]. It was reported that Methyl Vanillate@ZIF-8 immobilized on titanium scaffold sustainably released the Zn^2+^ and Methyl Vanillate for preferable osteogenesis [44]. Recently, the angiogenic inducibility of the scaffolds modified with MOF crystals has also been reported [21, 45]. It was reported that the Cu-TCPP nanosheets interface endowed the scaffold with angiogenesis activity [45]. According to the findings of Chang’s study, Cu-based MOF integrated with exosome was developed on the stent for vascularization in regenerated bone [19]. In our study, the MIL-53(Fe) modification was confirmed to endow the titanium scaffolds with good biocompatibility (Fig. 4 & Fig.S1), pro-osteoblastogenesis and pro-angiogenesis in vitro (Figs. 5 and 6) and in vivo (Figs. 7 and 8). The excellent biocompatibility may be attributed to the chemical stability of MIL-53(Fe). Given that almost no iron ion released from MIL-53(Fe) [24, 25], the impact of iron ion on osteogenesis and angiogenesis induced by the MIL-53(Fe) coating was excluded. Furthermore, the “breathing” feature, the prominent characteristic of MIL-53(Fe), is considered to potentially play an important role. Our study provides a promising way of utilizing MOFs to activate the surface of titanium and satisfy the requirement of promoting sufficient vascularization in large bone defect reconstruction.

Driven by pro-angiogenic signals, the ECs transfer their identities into two specialized cell types, tip or stalk cells. Tip cells extend multiple filopodia, migrate forward, and form cell junctions with neighboring tip cells to develop vessels. Stalk cells, proliferating at the base of the sprouting vessel, participate in the formation of vascular lumens [9]. With a specific biomarker of *CD34* [46], tip cells are driven by the binding of the vital endothelial ligand, VEGF-A, and its corresponding receptor KDR [9]. The activation of KDR will significantly upregulate the expression of the *DLL4*, which in turn mediates the activation of *NOTCH* signaling to suppress the tip cell pattern in the trailing stalk ECs [9, 35]. *NOTCH* signaling can be strengthened by the heteromers forming by ID proteins and HES-1 proteins in stalk cells [47]. According to the findings proposed by Liu et al., activation of endothelial tip cells by electrochemically derived nanographene oxide was beneficial to accelerated angiogenesis in bone regeneration [4]. However, little research has concerned the effect of artificial bone repair scaffolds on the activation of tip cells. In this study, the expression profiles of tip-cell-related genes (*KDR*, *CD34* and *DLL4*) and stalk-cell-related genes (*HES-1*, *NOTCH1*, *ID-1* and *ID-2*) were evaluated in the HUVECs incubated in different groups. It was suggested that the MIL-53(Fe)@AHT groups have access to activating the tip cell phenotype at the early angiogenesis phage (Fig. 6). Collectively, MIL-53(Fe) coating enhanced accelerated vascularization in regenerated bone through activating tip cells.

Currently, most modifications of titanium scaffolds using MOF materials rely on the MOFs’ own degradation to release of functional ions, or the release of loaded drugs or vesicles [19, 21, 22, 34, 41]. MIL-53(Fe) possesses unique “breathing” characteristics due to its flexible structure, allowing it to regulate pore size and promote its adsorption of guest molecules [26]. Our research aims to utilize the “breathing” characteristics of MIL-53(Fe) to explore its natural induction of interactions with ECM components, thereby promoting interactions between the scaffold and cell. The dialogue between the scaffold and cell is conducive to promoting cellular mechanotransduction process, regulating cellular biological behaviors and gene expression. Some proteins in the ECM can promote the cellular mechanotransduction process, including laminin, fibronectin, and perlecan [48]. Laminin, as the main component of ECM and the binding motif of adhesion ligands, adjusted cytoskeleton re-organization via the mechanotransduction process [48]. Fibronectin, assembled into viscoelastic fibrils, has unique mechanical properties and acts as altering mechanotransduction signals sensed and transferred by cells [49]. Perlecan, a basement membrane heparan sulfate proteoglycan, serves as a repository of pro-angiogenic growth factor. More importantly, it interacts with collagen VI and XI to define and stabilize a pericellular matrix compartment, the mechanical cues from which will induce intracellular mechanotransduction [48]. It was demonstrated in our study that the MIL-53(Fe) modification allowed the titanium scaffold to dramatically increase the adsorption of these ECM proteins (Fig. 9A & S4), which implied the MIL-53(Fe) coating contributed cell mechanotransduction process. It was reported that the protein adsorption capacity of a scaffold was closely related to its wettability and roughness [50]. In this study, the micro-scale roughness among the scaffolds from different groups was similar, while the nanoscale roughness showed significant variation. The MIL-53(Fe)@AHT-1/2 group exhibited the optimal protein adsorption effect, which may be the comprehensive effect of wettability, nanoscale roughness, and the loading amount of MIL-53(Fe).

Biomechanical factors serve as triggers of vascular growth. ECs, as vital functional cells constituting blood vessels, possess mechanosensing capability and respond to mechanical stimuli through the mechanotransduction process [36]. The mechanotransduction mechanism allows ECs to alter their morphology, gene expression and cellular behavior, releasing inflammatory mediators and vasodilators, thereby influencing vascular formation [36]. Several studies have reported the use of mechanical cues to develop biomaterials for regulating vessel formation. It was indicated that the microenvironment topography cues could guide the phenotypes of ECs and vessel formation [51]. Biomaterial scaffolds, on the basis of optimizing the cell-interfaces to enhance focal adhesion, contribute to recruiting ECs and vascularization [52]. The mechanotransduction pathways include the integrins, cytoskeleton and intracellular signaling molecules [36]. The mechanotransduction signaling mediating the activation of vascular endothelial tip cell mainly include the integrin signaling, the G protein-coupled receptor signaling, and the hippo signaling [4, 13, 36]. Integrins, the transmembrane receptors composed of α and β subunits, link the ECM with the intracellular actin cytoskeleton. Integrin-based adhesion sites mediate the reaction to the biophysical cues of ECM. Subsequently, adhesive proteins such as vinculin are recruited to the adhesion sites [36]. In this study, the expressions of *Itg β1*, *Itg β3* and *Itg α5*, the integrins molecules specific to the mechanosense of ECs [13], were confirmed to be elevated in the HUVECs cultivated in the MIL-53(Fe)@AHT group. In addition, it was also suggested that the expression of *vinculin* was increased (Fig. 9). Focal adhesions (FAs), the intracellular part of which connecting integrins with the actin cytoskeleton, are the main components bridging the ECM and cells. Tyrosine phosphorylation FAK, which is distributed at the FAs of adherent cells, is the first event responding to the integrin-mediated cellular adhesion [14]. According to the findings of our study, the MIL-53(Fe) modification promoted the phosphorylation of FAK of HUVECs (Fig. 9F-G). Moreover, the hippo pathway is the crucial cascade that adjusts the intracellular mechanotrasduction [53]. The phosphorylation of *YAP***/***TAZ*, the core transcription factor in hippo signaling, blocks its nuclear translocation and thus inhibits the target gene expression. *RhoA* and *ROCK1*, the members of the Ras superfamily of GTPases, regulate cell shape changes via cytoskeleton re-organization as well as serve as master regulators of the mechanotrasduction process [53, 54]. The reduced phosphorylation of YAP, and elevated expression of RhoA and ROCK1 were confirmed in HUVECs co-cultured in MIL-53(Fe) modified scaffold. Consequently, the mechanotrasduction process of the vascular endothelial cell was activated by MIL-53(Fe) coating. This result should be attributed to its optimal extracellular matrix protein adsorption capacity. Similarly, several studies have reported the effect of the mechanotransduction mechanisms on promoting angiogenesis and activation of endothelial tip cells. The study from Liu et al. confirmed that the hippo-*YAP* signaling modulated by the nanographene oxide induced activation of tip cells and promoted angiogenesis [4]. It was demonstrated that the phospho-paxillin (*p-PXN*) - Rac Family Small GTPase 1 (*Rac1*) -*YAP* axis regulated by matrix stiffness mediated the tip cell formation [13]. Our study provided another strategy for the activation of tip cells and enhanced angiogenesis by facilitating the mechanotransduction process.

Cellular stiffness is a tangible manifestation characteristic of cellular mechanotransduction processes [13]. The cholesterol content of the cell membrane and the underlying actin cortex are the two main factors affecting cell stiffness. The reduced cholesterol content of the cell membrane or more oriented organization of the actin cortex induced increased cell stiffness [16, 17]. It was revealed that the MIL-53(Fe) modified scaffold could increase the cell stiffness of vascular endothelial cells (Fig. 10A). It was also confirmed by the phenomenon that the decreased cholesterol content of cell membrane and increased oriented organization of F-actin of ECs in MIL-53(Fe) modification group (Fig. 10C-D). The cell fate decision or cell phenotype transformation can be affected by cell stiffness [55–57]. For example, it was reported that the increased cell stiffness guided mesenchymal stem cells to differentiate into the osteogenic lineage. It has been indicated that the increased cell stiffness induced the endothelial tip cell formation [13]. In our study, the effect of cell stiffness on activation of tip cell and angiogenesis by MIL-53(Fe) coating was also confirmed by the rescue experiments (Fig. S6 & S7). The preliminary research of this study indicated that the scaffold’s regulation of cell stiffness of EC was adjusted by the mechanotransduction process, which included the integrin signaling, the G protein-coupled receptor signaling and the hippo signaling. The results of molecular docking simulations hinted that *RhoA*/*ROCK* in the G protein-coupled receptor signaling may play a crucial part in this process. Consequently, the MIL-53(Fe) coating has access to activating the tip cell phenotype and promoting angiogenesis by enhancing the cell stiffness of vascular endothelial cells.

There were some limitations in this research. Firstly, due to the limitation of cell mechanical detection technology, it was difficult to evaluate the cell stiffness of vascular endothelial cells seeded on the porous Titanium alloy scaffolds. The vascular endothelial cells for cell stiffness detection were incubated with conditioned medium collected from the indirect co-culture system of cells and substrates. Secondly, future studies of specific molecular mechanisms underlying cell stiffness variation of ECs controlled by MIL-53(Fe) coating were necessary.

## Conclusions

In summary, a biofunctionalized porous titanium scaffold with MIL-53(Fe)@AHT coating was developed in our study. It was identified that the “breathing” property of MIL-53(Fe) allowed the titanium scaffold to increase the adsorption of ECM proteins dramatically and thus facilitated the interaction between the scaffold and vascular endothelial cells. Furthermore, our results demonstrated that the intensive interaction between the cell and scaffold induced the mechanotransduction process and increased the cell stiffness of ECs, which further contributed to the activation of endothelial tip cells and promoted angiogenesis. As a result, the MIL-53(Fe) modified porous titanium scaffold enhanced the accelerated and sufficient vascularization in bone regeneration. Our study provided a novel strategy for titanium scaffold design with MOF nanomaterials to improve vascularization. Additional in-depth studies of the interaction between cell and scaffold from the perspective of manipulating cell stiffness will provide new ideas for scaffold development.

### Electronic supplementary material

Below is the link to the electronic supplementary material.

Supplementary Material 1

## Data Availability

No datasets were generated or analysed during the current study.
