# Supplementary material for: Modulating cell stiffness for improved vascularization: leveraging the MIL-53(fe) for improved interaction of titanium implant and endothelial cell
Source: J Nanobiotechnology. 2024 Jul 17;22:422. doi: 10.1186/s12951-024-02714-y (PMC11253409; doi:10.1186/s12951-024-02714-y)
Supplement: Supplementary file 1 — Supplementary Material 1 [file 12951_2024_2714_MOESM1_ESM.docx]

**Supporting information**

**Modulating Cell Stiffness for Improved Vascularization: Leveraging the MIL-53(Fe) for Improved Interaction of Titanium Implant and Endothelial Cell**

Jie Wu ^1, 2^, Leyi Liu ^1, 2^, Weidong Du ^1, 2^, Yunyang Lu ^1, 2^, Runze Li ^1, 2^, Chao Wang ^1, 2^, Duoling Xu ^1, 2^, Weili Ku ^1, 2^, Shujun Li ^3^, Wentao Hou ^3^, Dongsheng Yu ^1, 2*^, Wei Zhao ^1, 2*^

^1^ Hospital of Stomatology, Guanghua School of Stomatology, Sun Yat-sen University, Guangzhou, 510055, China

^2^ Guangdong Provincial Key Laboratory of Stomatology, Sun Yat-sen University, Guangzhou, 510050, China

^3^ Institute of Metal Research, Chinese Academy of Sciences, Shenyang, 110016, China

*Corresponding authors.

E-mail address:

zhaowei3@mail.sysu.edu.cn (Wei Zhao);

yudsh@mail.sysu.edu.cn (Dongsheng Yu).

Jie Wu, Leyi Liu and Weidong Du contributed equally to this work.

Table S1 The qPCR Primer Sequences

| **Gene** | **Primer** | **Sequences (5’-3’)** |
| --- | --- | --- |
| RUNX2 | Forward | GCGGTGCAAACTTTCTCCAG |
|  | Reverse | TCACTGCACTGAAGAGGCTG |
| Osterix | Forward | GGGGAAAGGAGGCACAAAG |
|  | Reverse | GTGAGGGAAGGGTGGGTAGTC |
| ALP | Forward | AGCGACACGGACAAGAAGC |
|  | Reverse | GGCAAAGA CCGCCACATC |
| OPN | Forward | GACAGCAACGGGAAGACC |
|  | Reverse | CAGGCTGGCTTTGGAACT |
| OCN | Forward | GAGGGCAGTAAGGTGGTGAA |
|  | Reverse | CGTCCTGGAAGCCAATGTG |
| KDR | Forward  Reverse | GGAAATCATTATTCTAGTAGGCACGACG  CCTGTGGATACACTTTCGCGATG |
| CD34 | Forward | GCCACCAGAGCTATTCCCAAA |
|  | Reverse | ATAAGGGTCTTCGCCCAGC |
| DLL4 | Forward | AAGAATGGGGCAACGTGCT |
|  | Reverse | GCCATCCTCCTGGTCCTTACA |
| HES-1 | Forward | ACGACACCGGATAAACCAAA |
|  | Reverse | CCGCGAGCTATCTTTCTTCA |
| NOTCH1 | Forward | GACTGCTCCCTCAACTTCAAT |
|  | Reverse | GTGGTCCTGCAGTACTGGT |
| ID-1 | Forward | GCTGCTCTACGACATGAACG |
|  | Reverse | CCAACTGAAGGTCCCTGATG |
| ID-2 | Forward | GTGGCTGAATAAGCGGTGTT |
|  | Reverse | TGTCCTCCTTGTGAAATGGTT |


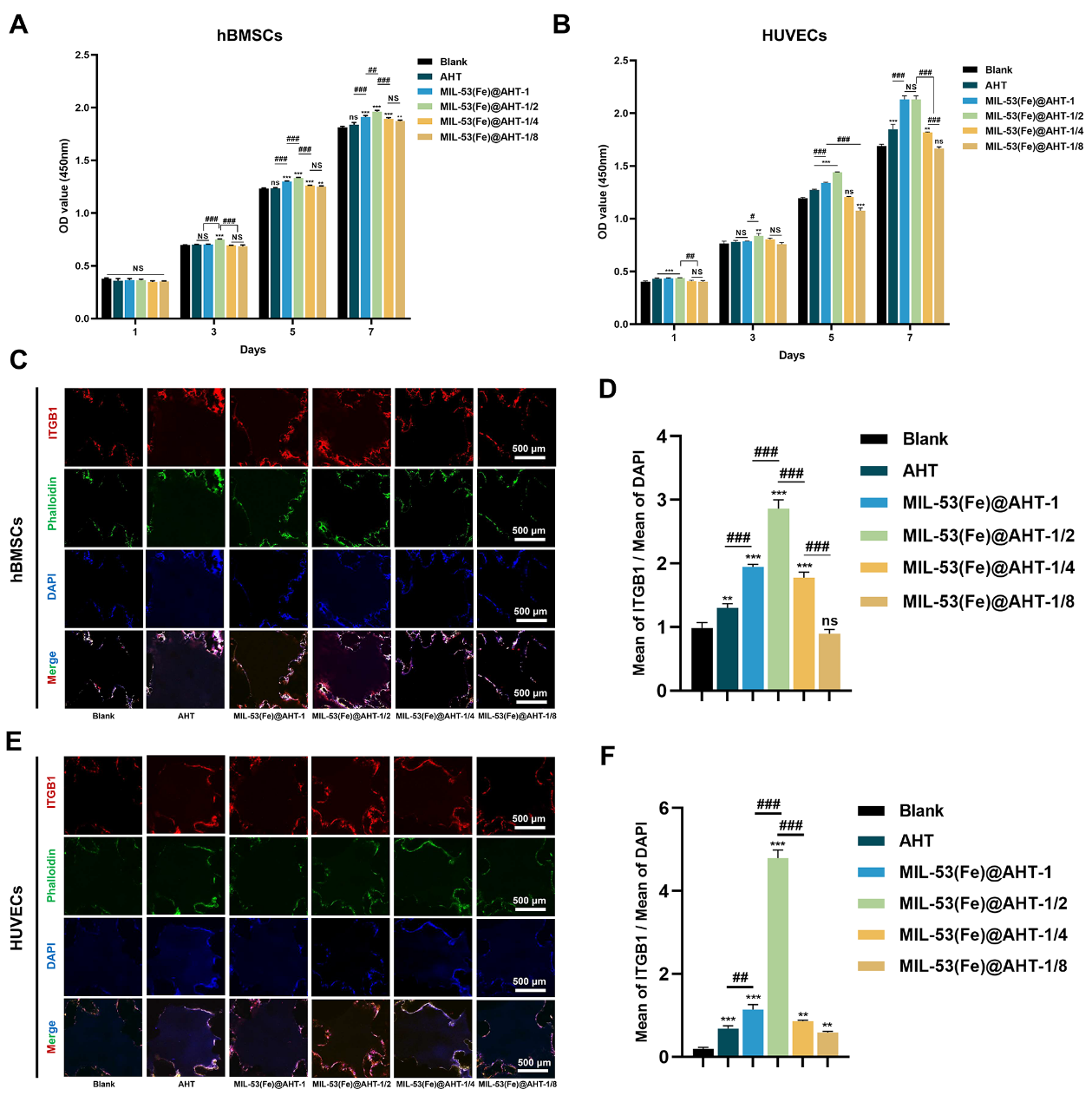


Fig. S1. Cell proliferation and adhesion of the hBMSCs and HUVECs seeded on the scaffolds. (A) CCK-8 assay of hBMSCs and (B) HUVECs on various scaffolds for 1, 3, 5 and 7 days. (C) The fluorescent images of hBMSCs cultivated on the scaffolds for 24 h. Scale Bar = 500 μm. (D) Semi-quantification analysis on ITGB1 expression of hBMSCs. (E) The fluorescent images of HUVECs seeded on the samples for 24 h. Scale Bar = 500 μm. (F) Semi-quantification analysis on ITGB1 expression of HUVECs. (n=3; ^ns^*P* > 0.05, ^*^*P* < 0.05, and ^**^*P* < 0.01, ^***^*P* < 0.001 compared with Ctrl group; ^NS^*P* > 0.05, ^#^*P* < 0.05, and ^##^*P* < 0.01, ^###^*P* < 0.001 compared among groups).


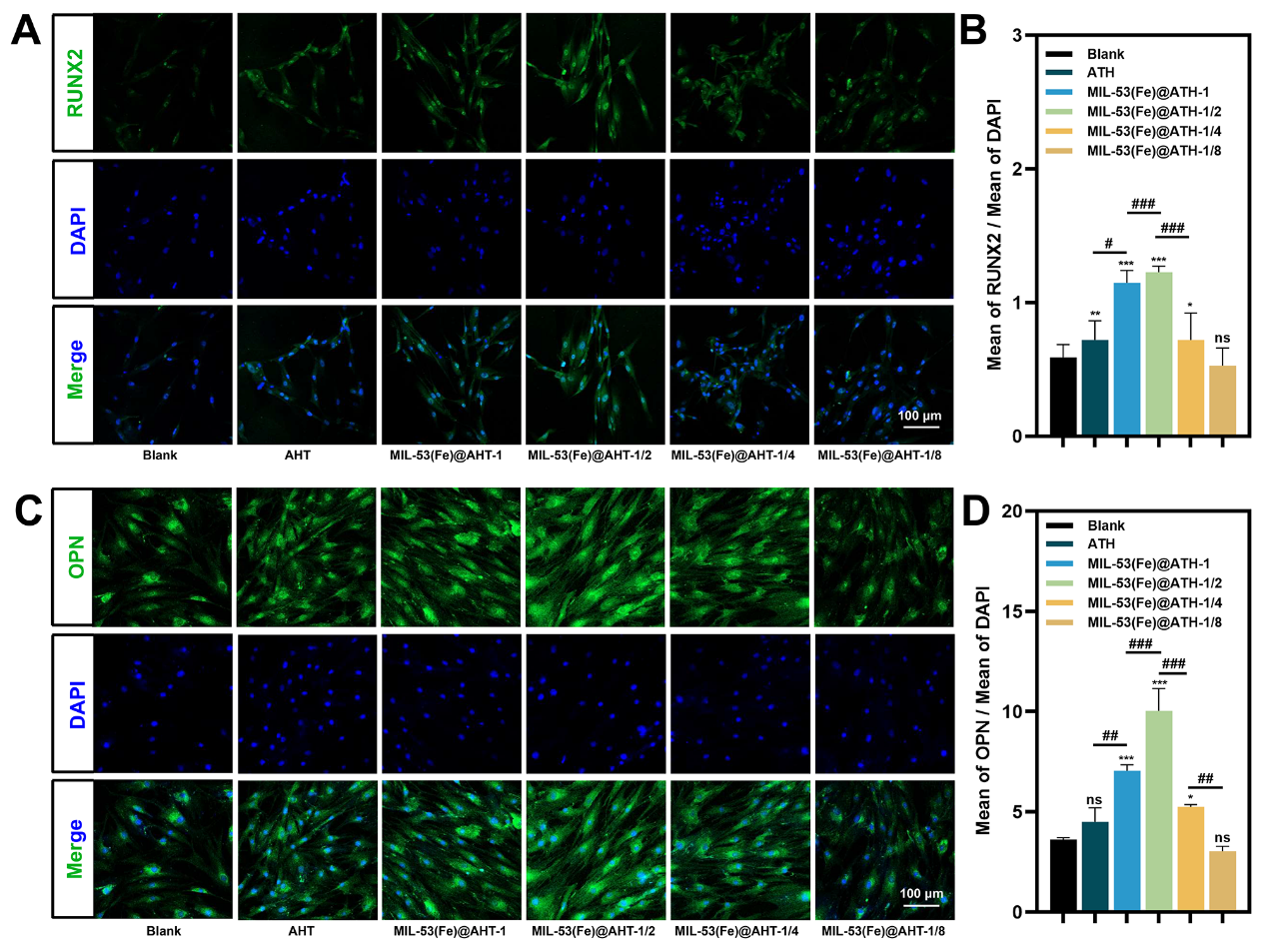
Fig. S2. Evaluation of osteogenic differentiation of hBMSCs using immunofluorescent staining. (A) Representative immunofluorescent images for RUNX2 expression of hBMSCs cultivated in conditioned medium from different samples and (B) the corresponding quantitative analysis. (C) Representative immunofluorescent images for OPN expression of hBMSCs cultivated in conditioned medium from different samples and (D) the corresponding quantitative analysis. (n=3; ^ns^*P* > 0.05, ^*^*P* < 0.05, and ^**^*P* < 0.01, ^***^*P* < 0.001 compared with Ctrl group; ^NS^*P* > 0.05, ^#^*P* < 0.05, and ^##^*P* < 0.01, ^###^*P* < 0.001 compared among groups).


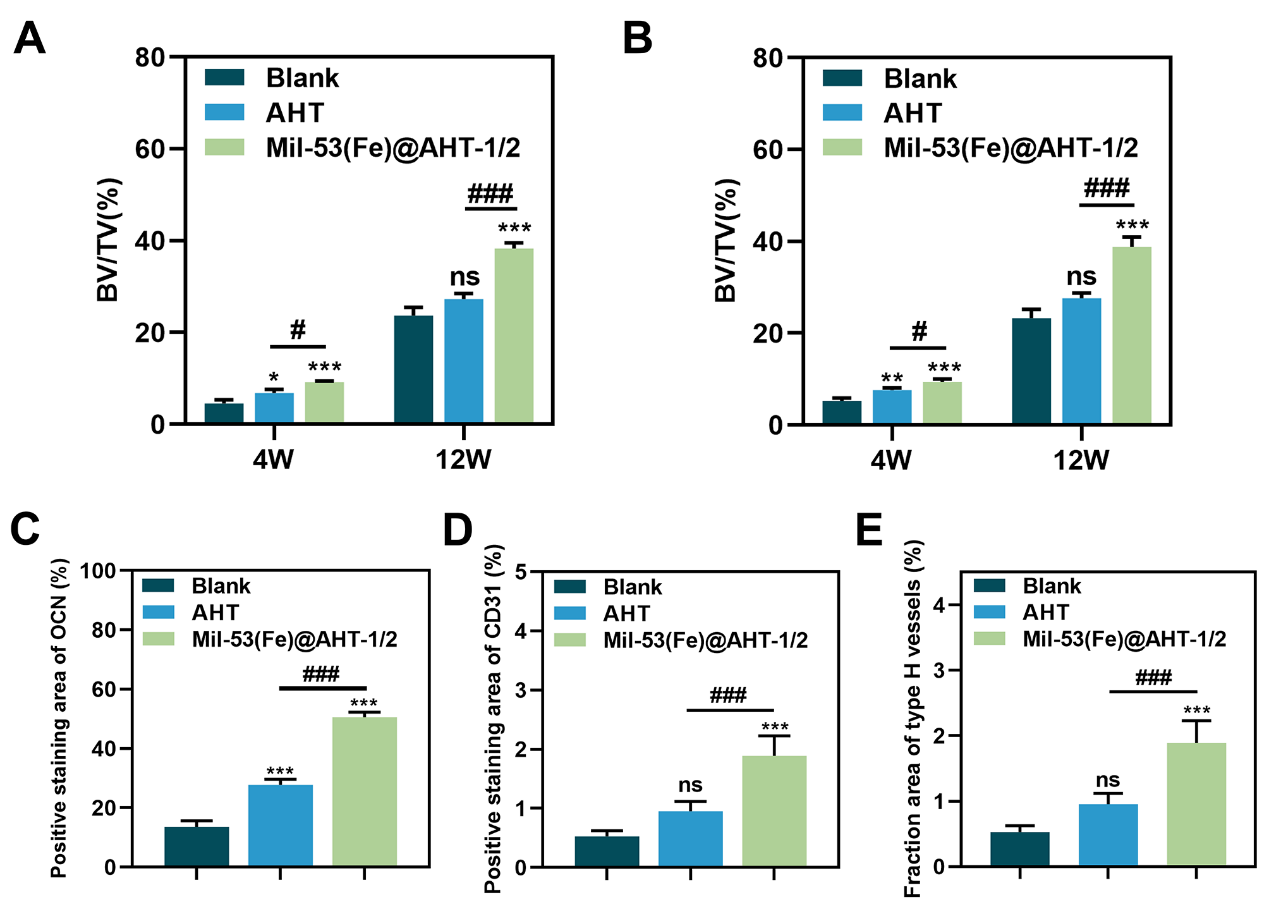


Figure S3. Quantitative analysis of the H & E staining, Goldner's Trichrome staining, immunohistochemical and IF staining on vascularized bone regeneration after 4 weeks or 12 weeks. (A) Quantitative analysis of H & E staining. (B) Quantified analysis of Goldner's Trichrome staining. (C) quantified analysis of the positive area of the immunohistochemical staining images of OPN and (D) CD31. (E) Quantified analysis of the fraction area of type H vessels.


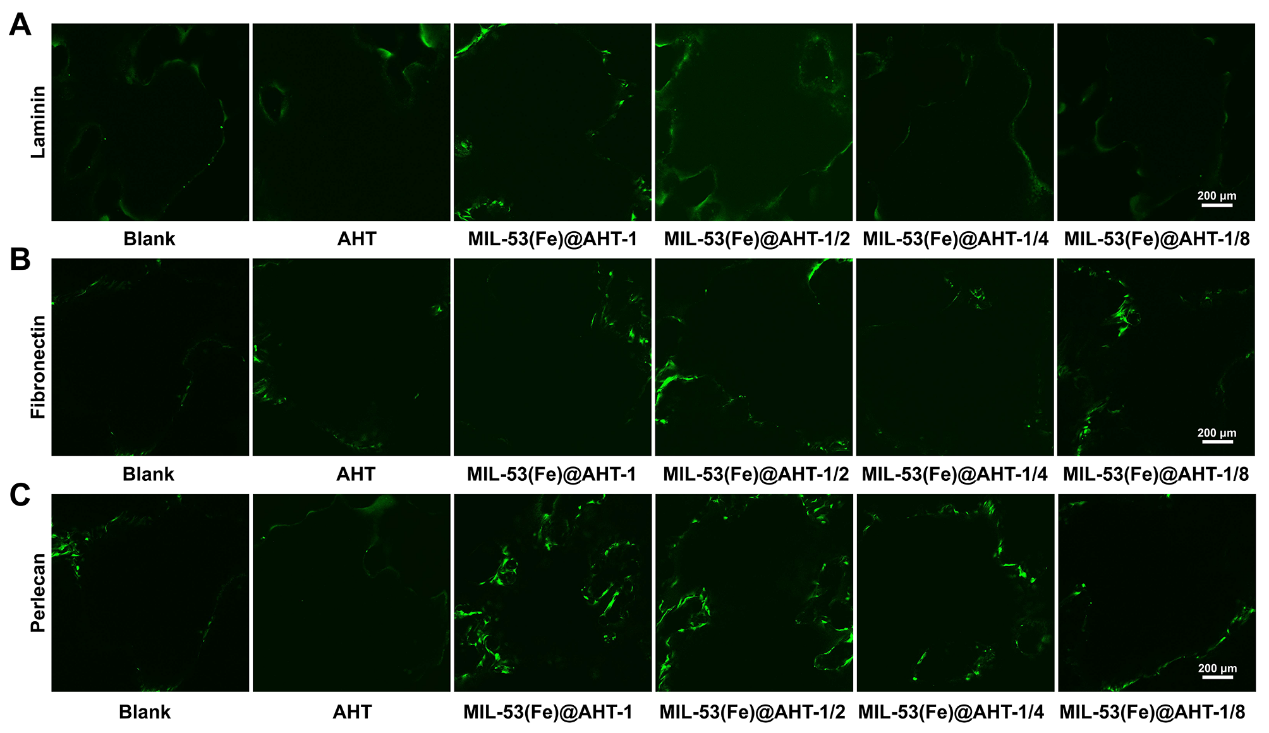


Fig. S4. Representative fluorescent images of absorption in (A) laminin, (B) fibronectin and (C) percelan on different scaffolds. Scale Bar = 200 μm.


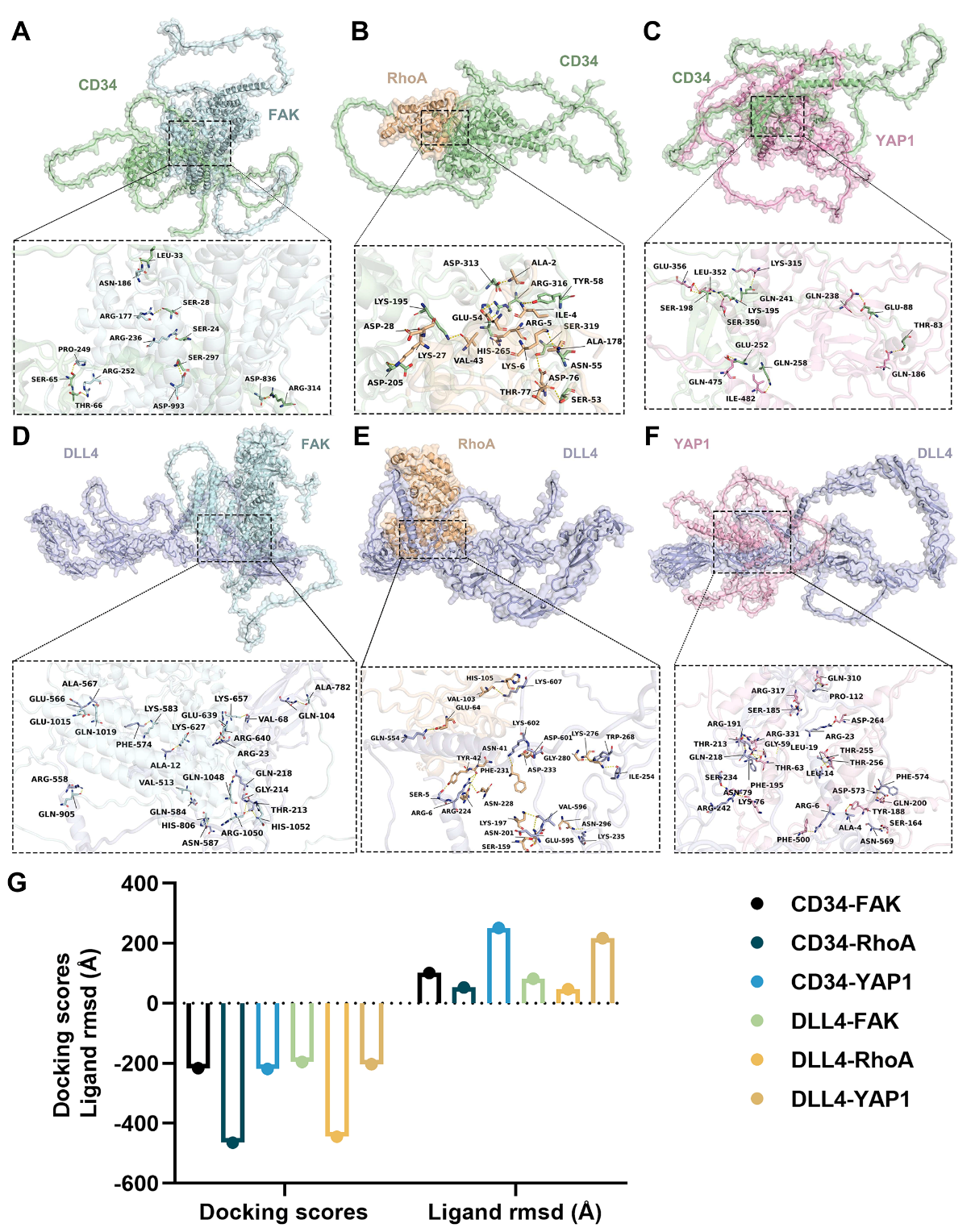


Fig. S5. Molecular docking simulation between protein CD34 or DLL4 with FAK, RhoA or YAP1. Binding interaction geometries between CD34 with (A) FAK, (B) RhoA or (C) YAP1, and between DLL4 with (D) FAK, (E) RhoA or (F) YAP1. (G) The corresponding docking scores and ligand rmsd (Å).


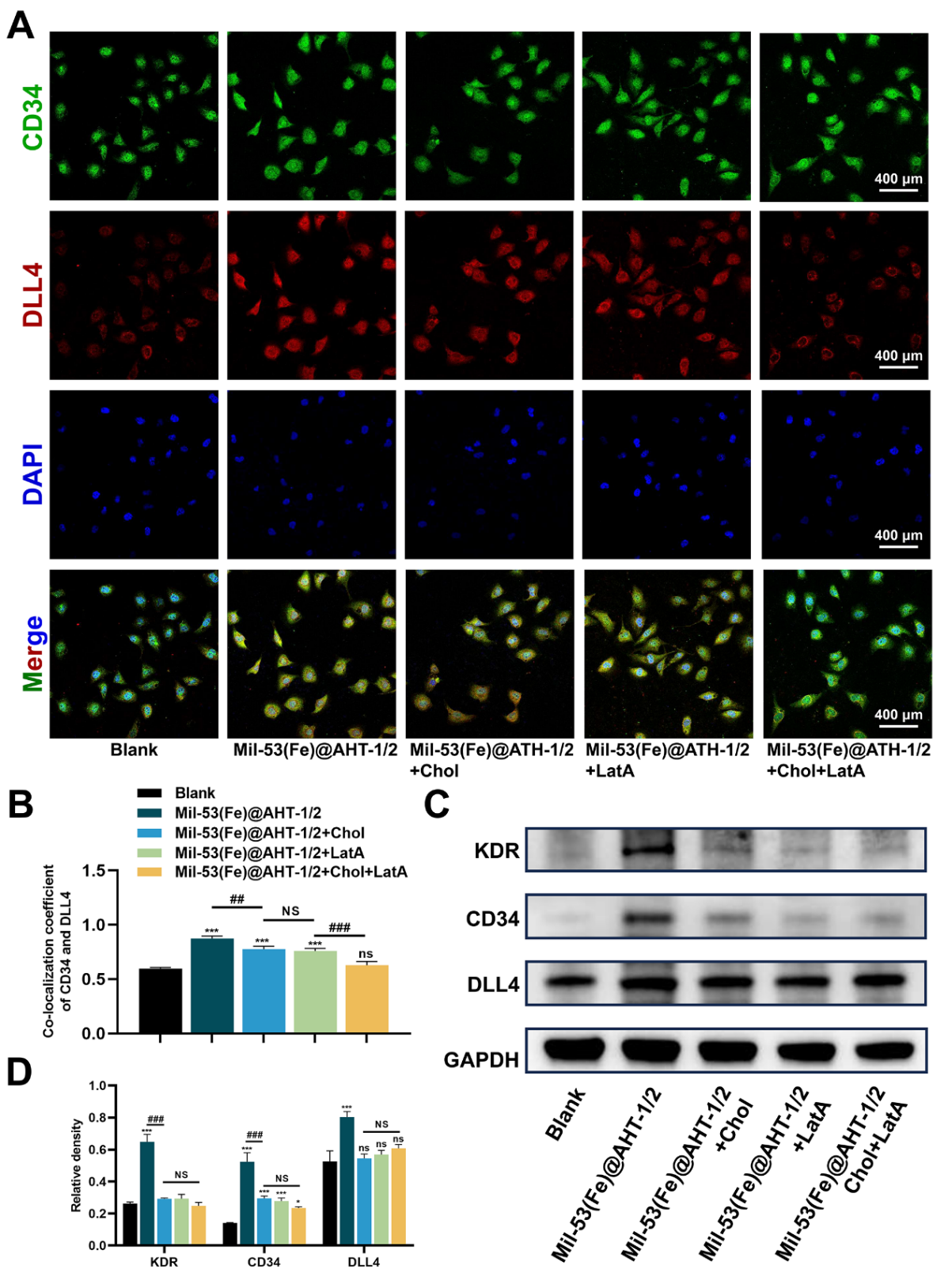


Fig. S6. The effect of cell stiffness on endothelial tip cell activation by the scaffolds. (A) Immunofluorescence staining images showing CD34/ DLL4 positive cells in different groups. Scale bar = 400 μm. (B) The quantified analysis of the correlation of CD34 and DLL4 in different groups. (C) Immunoblotted images for KDR, CD34, DLL4 and GAPDH of HUVECs of diverse groups and (D) the corresponding quantified analysis on protein levels. (n=3; ^ns^*P* > 0.05, ^*^*P* < 0.05, and ^**^*P* < 0.01, ^***^*P* < 0.001 compared with Ctrl group; ^NS^*P* > 0.05, ^#^*P* < 0.05, and ^##^*P* < 0.01, ^###^*P* < 0.001 compared among groups).


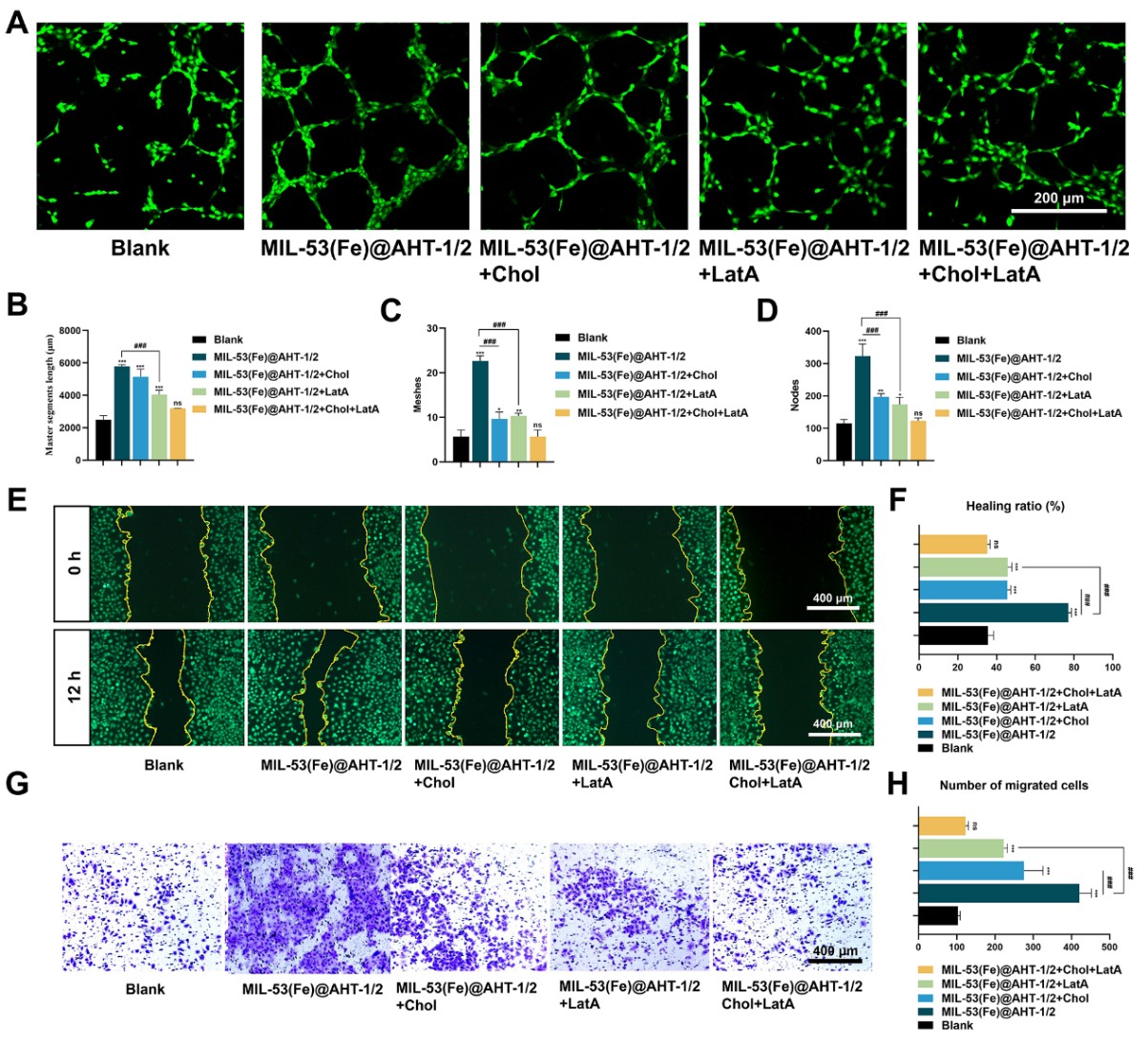


Fig. S7. The effect of cell stiffness on angiogenesis by the scaffolds. (A) Representative images of the tube formation and quantified evaluation of (B) master segments length, (C) meshes and (D) nodes of HUVECs cultivated in sample conditioned medium from Blank group and MIL-53(Fe)@AHT-1/2 group, the sample conditioned medium supplemented with either cholesterol or LatA or both, respectively (MIL-53(Fe)@AHT-1/2 + Chol, MIL-53(Fe)@AHT-1/2 + LatA and MIL-53(Fe)@AHT-1/2 + Chol + LatA). Scale bar = 200 μm. (E) Wound healing assay of HUVECs cultivated in different sample conditioned medium and (F) the quantified analysis on wound healing area (%). Scale bar = 400 μm. (G) The microscopic images and (H) the quantified numbers of the migrated cells induced by different sample conditioned mediums in transwell assay for 24 h. Scale bar = 400 μm. (n=3-5; ^ns^*P* > 0.05, ^*^*P* < 0.05, and ^**^*P* < 0.01, ^***^*P* < 0.001 compared with Ctrl group; ^NS^*P* > 0.05, ^#^*P* < 0.05, and ^##^*P* < 0.01, ^###^*P* < 0.001 compared among groups).
